# Enhancing the antimycobacterial efficacy of pyridine-4-carbohydrazide: linkage to additional antimicrobial agents *via* oxocarboxylic acids†

**DOI:** 10.1039/d4md00663a

**Published:** 2024-10-16

**Authors:** Václav Pflégr, Klára Konečná, Jiřina Stolaříková, Jan Ősterreicher, Ondřej Janďourek, Martin Krátký

**Affiliations:** a Department of Organic and Bioorganic Chemistry, Faculty of Pharmacy in Hradec Králové, Charles University Akademika Heyrovského 1203 500 03 Hradec Králové Czech Republic martin.kratky@faf.cuni.cz +420 495067166 +420 495067302; b Department of Biological and Medical Sciences, Faculty of Pharmacy in Hradec Králové, Charles University Akademika Heyrovského 1203 500 03 Hradec Králové Czech Republic; c Laboratory for Mycobacterial Diagnostics and Tuberculosis, Regional Institute of Public Health in Ostrava Partyzánské náměstí 7 Ostrava Czech Republic

## Abstract

This study evaluates the antimycobacterial potential of novel “mutual” bioactive amides, combining pyridine-4-carbohydrazide (isoniazid, INH) with various antimicrobial agents (sulphonamides, 4-aminosalicylic acid, thiosemicarbazide, diphenyl (thio)ethers) *via* oxocarboxylic acids. The aim was to enhance activity against both drug-susceptible and multidrug-resistant (MDR) *Mycobacterium tuberculosis* and non-tuberculous strains, while overcoming drug resistance through dual-action mechanisms. Many derivatives exhibited potent antimycobacterial activity, with minimum inhibitory concentrations (MICs) as low as ≤0.25 μM, outperforming INH, especially diphenyl (thio)ethers and biphenyl analogues. Additionally, the compounds were effective against *M. kansasii* (MICs ≤1 μM) and inhibited MDR strains at higher concentrations (≥8 μM). The cytotoxicity assay indicated a favourable safety profile, with no significant haemolysis at 125 μM, and some compounds were even protective. Selectivity for mycobacteria was confirmed by low inhibition of Gram-positive bacteria and inactivity against Gram-negative bacteria or fungi, highlighting the potential for further development as antimycobacterial agents.

## Abstract 2

Linking pyridine-4-carbohydrazide to other antimicrobial agents *via* oxocarboxylic acids significantly enhances efficacy against mycobacteria with promising selectivity and safety profiles.

## Graphical abstract

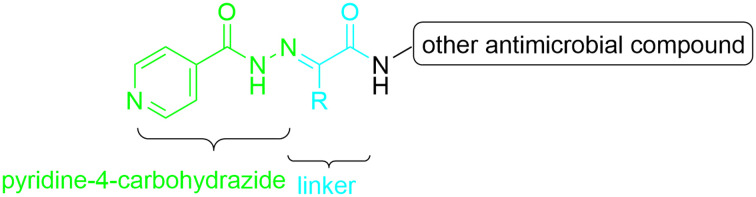

## Introduction

Tuberculosis (TB) is a severe infectious disease caused by mycobacteria from the *Mycobacterium tuberculosis* complex, most commonly the *Mycobacterium tuberculosis* (*Mtb*) strain. The disease in its pulmonary form mainly affects weakened, malnourished, immunocompromised, and sick people, more men than women, and fewer children than adults. Although the incidence of tuberculosis in developed countries has been decreasing year by year, in the global context, TB is one of the leading causes of death from a single infectious agent (ranking above HIV/AIDS). TB mortality was surpassed only by the SARS-CoV-2 virus (COVID-19 pandemic in 2019–2022), according to the number of reported cases.^1^ Another equally dangerous group is non-tuberculous (atypical) mycobacteria (NTM), which includes many agents that can be potentially pathogenic for humans (*M. kansasii*, *M. avium* complex, *M. abscessus*, *M. xenopi*, and many others).^2^ The clinical symptoms of the mentioned mycobacterioses (especially in the case of pulmonary forms) are often mistaken for TB, also because the most at-risk group are people with the same problems as in the case of TB. Unlike tuberculosis, the incidence of atypical mycobacterioses shows an increasing trend in reported cases. Unfortunately, treating non-tuberculous mycobacterioses is much more complicated than treating TB. Thus, the development of antimycobacterial drugs affecting NTM is also essential.^3–5^

Two of the viable approaches to obtain novel antimicrobial agents are the mutual prodrug (co-drug) strategy and molecular hybridization. They can offer significant advantages in efficacy and pharmacokinetic issues.^6^ Mutual prodrugs combine two bioactive drugs that synergistically enhance therapeutic effects while minimizing their drawbacks, such as toxicity or acquired resistance development. In fact, they are a combination of two drugs in one molecular entity. Regarding increasing resistance, the potential of mutual prodrugs to combat resistant pathogens effectively has been highlighted.^7,8^ For instance, a co-drug combining isoniazid (INH) with α-lipoic acid exhibited *in vivo* the same level of antimicrobial activity as the parent INH, while demonstrating reduced toxicity.^9^ Co-drugs combining pyrazinoic acid and the salicylanilide scaffold through ester bonds significantly enhanced the antimycobacterial activity of both components, including efficacy against resistant strains.^10^ Molecular hybridization in the research of anti-infective drugs integrates diverse molecular fragments to obtain new non-cleavable compounds with superior pharmacological properties. Combining different pharmacophores enables the development of compounds, again, especially with enhanced potency against infectious agents. This approach has been widely and successfully applied, *e.g.*, for fluoroquinolone drugs.^7,11,12^

**Fig. 1:**
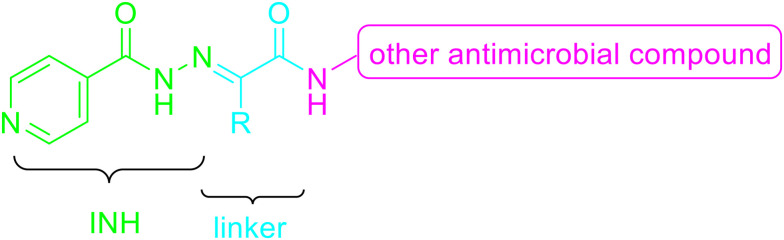
General structure of targeted derivatives.

These facts lead us to investigate new “me-too” derivatives of INH using the co-drug approach to describe dually active derivatives of this clinically crucial antituberculosis drug. (*E*)-2-(2-Isonicotinoylhydrazineylidene)propanoic acid was used as the starting material that was modified *via* amide formation at the free carboxyl group. The advantages of using this starting material to obtain new promising compounds have been described in detail previously, for instance, synthetic versatility and non-toxicity of the linker.^13,14^ In these studies, we demonstrated higher activity of INH conjugates with halogenated anilines^13^ and bioactive alcohols and phenols^14^ against *Mtb* as well as NTM, including partial activity against INH-resistant strains, mostly without toxicity. The predominant mechanism of action was identified as InhA inhibition, although it was not the only one. However, this is a little-explored molecular space in which INH is attached to a bifunctional oxocarboxylic acid linker, which opens the way for many other structural modifications. Thus, we used this strategically designed bifunctional linker to couple the INH scaffold with another antimycobacterial active compound to improve antimycobacterial potency against both TB and NTM. Developing new, safe, and effective treatments for *Mycobacterium tuberculosis* and NTM continues to be a challenging goal.^15^ The design of targeted compounds is depicted in Fig. 1.

## Experimental

### General

All chemicals used for syntheses were purchased from Merck KGaA (Darmstadt, Germany), Penta Chemicals UnLtd. (Prague, Czech Republic), and Lach-Ner (Neratovice, Czech Republic) and were used as received without additional purification. The progress of reactions was monitored using thin-layer chromatography (TLC). Alugram SIL G/UV_254_ plates with a 0.2 mm layer of silica gel 60 F_254_ from Macherey-Nagel (Düren, Germany) were used as the stationary phase, and a mixture of dichloromethane and methanol in a volume ratio of 93 : 7 was used as the mobile phase. Silica gel “Kieselgel 60” (0.040–0.063 mm) from Merck KGaA (Darmstadt, Germany) was used for column chromatography. Solvents ranging from pure dichloromethane to a mixture of dichloromethane and methanol in a volume ratio of 93 : 7 were used as eluents for preparative column chromatography in a gradient arrangement. A UV lamp Camag (DonauLab, Prague, Czech Republic) emitting a wavelength of 254 nm was used for detection. Uncorrected melting points were recorded using a Büchi B-545 apparatus (Büchi Labortechnik AG, Flawil, Switzerland).

Structural identities of the prepared compounds were confirmed using ^1^H NMR and ^13^C NMR spectroscopy analysis. NMR spectra (500 MHz for ^1^H and 126 MHz for ^13^C) were measured in DMSO-*d*_6_ or CF_3_COOD as solvents at ambient temperature using a Varian VNMR S500 instrument (Varian, Palo Alto, CA, USA). The values of chemical shifts (*δ*) are given in parts per million (ppm), and the spectra were referenced internally to tetramethyl silane (TMS) as a standard by the residual signal of the protic solvent (DMSO-*d*_6_: 2.50 for ^1^H, 39.70 for ^13^C and CF_3_COOD: 11.5 for ^1^H, 116.5 and 164.4 for ^13^C). The coupling constants (*J*) are reported in Hz. NMR spectra were processed using the MestReNova program (Mestrelab Research, Santiago de Compostela, Spain). Infrared spectra were measured by the ATR technique using a germanium crystal with a Nicolet 6700 FT-IR spectrophotometer (Thermo Fisher Scientific, Waltham, MA, USA) in the range of 600–4000 cm^−1^. Elemental analysis was performed using a Vario MICRO cube element analyzer (Elementar Analysensysteme, Hanau, Germany), with results presented as percentage values. Yields are presented as the ratio of the isolated yield to the theoretical yield.

Images of chemical structures, schemes, and calculated constants were acquired using ChemDraw Professional, version 22.2.0.3300 (PerkinElmer Inc., Waltham, MA, USA).

### Procedure for synthesis of acids 2

According to our previously reported procedure, 2-(2-isonicotinoylhydrazineylidene)propanoic acid 2a was obtained with a yield of 98%.^14,16^ 2-(2-Isonicotinoylhydrazineylidene)acetic acid^17^2b was prepared similarly, replacing pyruvic acid with glyoxylic acid. Isoniazid (137 mg, 1.0 mmol) was dissolved in hot methanol (10 mL) with vigorous stirring. Then, 1.1 equivalents of glyoxylic acid monohydrate (101 mg, 1.1 mmol) were added in one portion. The reaction mixture was refluxed for 2 hours, during which the formation of a solid product was observed. After refluxing, the mixture was allowed to cool to room temperature and then stored at +8 °C for 1 hour. The formed precipitate was filtered off, washed with hot methanol (2 × 3 mL), and dried to yield pure acid 2b with a yield of 96%. Its physical and spectral characteristics were consistent with those reported in the literature.^17,18^

### Procedure for synthesis of amides

1.0 mmol of acid 2a (207 mg) or 2b (193 mg) was suspended in 10 mL of *N*,*N*-dimethylformamide (DMF). 1.0 mmol of the corresponding amine and 1.2 mmol of 1-hydroxybenzotriazole (HOBt; 162 mg) were introduced into the reaction vessel under constant stirring. The reaction mixture was cooled to 0 °C in an ice bath, and then 1.3 mmol of 1-ethyl-3-(3-dimethylaminopropyl)carbodiimide hydrochloride (EDC; 248 mg) was added in one portion. The reaction mixture was allowed to react overnight with gradual thawing of the ice bath. The solvent was removed under reduced pressure, and the resulting brown oily mass or crude solid products were dissolved/suspended in 25 mL of ethyl acetate, and then treated successively with 2 × 25 mL of saturated sodium bicarbonate solution and 25 mL of saturated brine solution (compounds 3h–3q and 4j). Sulphonamide derivatives 3a–3g, 4a, 4c, 4e and 4f were treated only with demineralised water (2 × 25 mL) and brine (1 × 25 mL). The organic phase was dried with sodium sulphate. The drying agent was filtered off, the ethyl acetate was evaporated under reduced pressure, and the crude product was purified by crystallization from aqueous acetonitrile or by gradient column chromatography.

The compounds containing an NH_2_ group used for the coupling with acids 2 included the following: sulfanilamide, sulfamethoxazole, sulfathiazole, sulfamethoxypyridazine, sulfadiazine, sulfamethazine, sulfameter, methyl 4-aminosalicylate, isoniazid, thiosemicarbazide, benzene-1,4-diamine, 2-phenoxyaniline, 4-phenoxyaniline, 4-(4-chlorophenoxy)aniline, 3-chloro-4-(4-chlorophenoxy)aniline, 4-[(4-nitrophenyl)thio]aniline, and [1,1′-biphenyl]-4-amine. All of them were commercially available.

The diamine-based derivative 3k was synthesized analogously by reacting 1.0 mmol of acid 2a (207 mg) with 0.5 mmol of benzene-1,4-diamine (54 mg), 1.2 mmol of HOBt (162 mg) and 1.3 mmol of EDC (248 mg).

The physical and spectral characterization data of the prepared compounds are attached in the ESI.†

### Biological activity

#### Antimycobacterial activity

The antimycobacterial activity of the target derivatives and bioactive precursors was assessed following a previously described method.^19^ In flat-bottom microtitration plates, minimum inhibitory concentrations (MICs) were determined using Šula's liquid semisynthetic medium (SEVAC, Prague, Czech Republic). The final volume in each well was 200 μL, consisting of 100 μL of the tested compound solution and 100 μL of the mycobacterial inoculum. The inoculum was prepared to achieve a cell density of 3 × 10^5^ CFU mL^−1^. Thus, the final bacterial load was 1.5 × 10^5^ CFU mL^−1^ per well. MIC values were determined visually. Compounds were dissolved in DMSO, and INH was dissolved in sterile distilled water and added to the medium, resulting in a final 1.0% DMSO (v/v) concentration that did not affect mycobacterial growth. The mycobacterial strains included the drug-sensitive *Mycobacterium tuberculosis* strain CNCTC 331/88 (Czech National Collection of Type Microorganisms; H37Rv; stock suspension dilution 10^−3^) and two non-tuberculous species: *Mycobacterium avium* ssp. *avium* CNCTC 330/88 (resistant to INH, rifampicin (RIF), rifabutin, ofloxacin (OFX), and ethambutol (EMB); dilution 10^−5^) and a clinical isolate of *Mycobacterium kansasii* (6509/96; dilution 10^−4^). MIC values were determined using a two-fold serial dilution method ranging from 250 to 0.125 μM and 1000 to 1 μM for *Mtb* and NTM, respectively. MIC [μM] represented the lowest concentration inhibiting mycobacterial growth completely after 14 and 21 days of incubation, with an additional 7 day assessment for *M. kansasii*. Precursor INH, a first-line antitubercular drug, was used as a reference drug for MIC comparison.

The most effective derivatives were further evaluated against seven drug-resistant TB strains (dilution 10^−3^), each exhibiting distinct resistance profiles. All strains were resistant to INH, RIF, rifabutin, and streptomycin (STM), with additional resistances noted: strain 7357/1998 was also resistant to EMB and OFX; strain 234/2005 to EMB; strain 8666/2010 to EMB, OFX, and clofazimine (CLO); strain Praha 1 to EMB and CLO; strain Praha 4 to EMB, OFX, and CLO (all MDR-TB); and strain Praha 131 to INH, rifamycins, STM, EMB, OFX, gentamicin (GEN), and amikacin (AMK). These are clinical isolates from patients examined at the Regional Institute of Public Health in Ostrava. MIC values for these resistant strains were determined *via* two-fold serial dilution ranging from 250 to 0.25 μM.

#### Antibacterial activity^20^

Screening of antibacterial activity for target compounds comprised evaluation against four Gram-positive and four Gram-negative bacterial strains of clinical significance. Specifically, these included *Staphylococcus aureus* ATCC (American Type Culture Collection) 29213, CCM (Czech Collection of Microorganisms) 4223; methicillin-resistant *Staphylococcus aureus* (MRSA) ATCC 43300, CCM 4750; *Staphylococcus epidermidis* ATCC 12228, CCM 4418; *Enterococcus faecalis* ATCC 29212, CCM 4224; *Escherichia coli* ATCC 25922, CCM 3954; *Klebsiella pneumoniae* ATCC 10031, CCM 4415; *Acinetobacter baumannii* ATCC 19606, DSM (Deutsche Sammlung von Mikroorganismen und Zellkulturen, German Collection of Microorganisms and Cell Cultures) 30007; and *Pseudomonas aeruginosa* ATCC 27853, CCM 3955. These strains were obtained from the CCM (Brno, Czech Republic) or DSM (Braunschweig, Germany).

The microdilution broth method was applied according to EUCAST instructions^21^ with slight modification in 96-well plates. The final volume of the cultivation medium containing the tested compound(s) at concentrations ranging from 0.98 μM to 500 μM, along with the bacterial suspensions, was adjusted to a total of 210 μL. This same volume was maintained for both positive and negative controls. The inoculum was prepared using cation-adjusted Mueller-Hinton broth (CA-MHB, M-H 2 Broth). Bacterial suspensions were made from 16–24 hour-old bacterial cultures, and their turbidity was measured. The inoculum was then adjusted with CA-MHB to reach a final cell density of approximately 1 × 10^6^ CFU mL^−1^. A volume of 10 μL of bacterial suspension was added to each well, except in the negative controls, where 10 μL of CA-MHB was used instead. The final bacterial load per well (except in the negative controls) was approximately 5 × 10^5^ CFU mL^−1^ (range: 3–7 × 10^5^ CFU mL^−1^), as recommended by EUCAST guidelines. This cell density corresponds to approximately 6 × 10^4^–1.5 × 10^5^ CFU per well. Compounds were dissolved in DMSO to prepare stock solutions. The final concentration of DMSO in the testing medium did not exceed 1.0% (v/v) and did not impact bacterial growth. All chemicals were purchased from Merck KGaA (Darmstadt, Germany).

Positive controls consisted of microbes alone, while negative controls comprised a medium with DMSO. Antibacterial activity was quantified as minimum inhibitory concentration (MIC, reported in μM) after 24 and 48 hours of static incubation in a dark, humidified atmosphere at 35 ± 2 °C. MIC was determined visually by the naked eye in the well containing the lowest drug concentration, where no visible microbial growth was registered.

Standard antibiotics (ciprofloxacin, gentamicin, and piperacillin, data not shown) and internal quality controls were included in antibacterial activity testing.

#### Antifungal activity^20^

Antifungal activity was assessed against four yeast strains: *Candida albicans* ATCC 24443, CCM 8320; *Candida krusei* ATCC 6258, CCM 8271; *Candida parapsilosis* ATCC 22019, CCM 8260; and *Candida tropicalis* ATCC 750, CCM 8264. Additionally, four strains of filamentous fungi were included: *Aspergillus fumigatus* ATCC 204305, *Aspergillus flavus* CCM 8363; *Lichtheimia corymbifera* CCM 8077; and *Trichophyton interdigitale* ATCC 9533, CCM 8377. The microdilution broth method was conducted following EUCAST guidelines^22,23^ with minor adjustments. The antifungal activity screening was conducted using 96-well microplates. The final volume of the cultivation medium, containing the tested compound(s) in a concentration range of 0.98 μM to 500 μM and fungal suspensions, was 210 μL per well. The same final volume was maintained for both the positive and negative controls. An RPMI-1640 medium, supplemented with 2% glucose (w/v) and buffered to pH 7.0 using 3-(*N*-morpholino)propane-1-sulphonic acid, was used to prepare the yeast inoculum. An 18–24 hour-old yeast culture was adjusted based on turbidity measurements and further diluted with the above-mentioned RPMI-1640 medium to achieve a final cell density of approximately 1–5 × 10^6^ CFU mL^−1^. A volume of 10 μL of the prepared yeast suspension was added to each well, except for the negative controls, where 10 μL of RPMI-1640 medium was used. The final yeast cell density per well (except for the negative controls) was approximately 1–5 × 10^5^ CFU mL^−1^, consistent with the EUCAST recommendations. This corresponds to approximately 2 × 10^4^ to 1 × 10^5^ CFU per well. Compounds under investigation were dissolved in DMSO to prepare stock solutions. All media and chemicals were purchased from Merck KGaA (Darmstadt, Germany). The final concentration of DMSO in the test medium did not exceed 1.0% (v/v), ensuring that it did not inhibit fungal growth.

Incubation was performed statically in a dark, humidified atmosphere at 35 ± 2 °C for 24 and 48 hours (72 and 120 hours for *T. interdigitale*). Positive controls comprised the fungus alone, while negative controls consisted of a medium with DMSO. MIC endpoints were determined visually, identifying the lowest drug concentration in wells where no visible microbial growth occurred.

Two commonly used standard antifungal drugs (amphotericin B and voriconazole; data not shown) were included as reference standards for testing antifungal activity against selected quality control yeast strains.

#### Determination of haemolytic activity using an *ex vivo* human red blood cell haemolysis assay

Human blood samples were obtained from informed donor volunteers in accordance with the Blood Collection Practice Guideline complying EU standards and directives. Experiments with human blood were approved by the Ethics Committee, University Hospital Hradec Králové, Czech Republic (Ethics Committee approval No. 202402 P01). Informed consent was obtained from human participants of this study.

Human blood samples were processed by centrifugation at 1000*g* for 10 minutes. After discarding the supernatants, the cell pellets were washed three times with Hartmann's solution. The final cell pellet was diluted 1 : 7 (v/v) in Hartmann's solution. Approved antitubercular drugs RIF and EMB (both Merck, USA), INH, bedaquiline, and pyrazinamide (all Thermo Fisher Scientific, USA), as well as tested compounds, were dissolved in DMSO (Merck, USA) to prepare stock solutions. Subsequently, compounds at desired concentrations in Hartmann's solution were prepared. A volume of 0.5 mL of the cell suspension was then mixed with 0.5 mL of the tested compound in Hartmann's solution (final concentration of DMSO 1.0%, v/v) and incubated at 37 °C for 1 hour. After incubation, the cell suspensions were centrifuged, and the supernatants were carefully collected. The release of haemoglobin from human red blood cells (hRBCs) into the supernatants was measured by absorbance at 405 nm^24^ using a spectrophotometer Synergy HTX multi-mode reader. Negative controls (hRBCs in Hartmann's solution with a final DMSO concentration of 1.0%, v/v) and positive controls (hRBCs in Hartmann's solution, sonicated with an ultrasonic processor (UP100H, Hielscher, Germany), for 1 minute) were included in each assay.

Statistical analysis was conducted using Graph-Prism software version 9.0.0 (GraphPad Software, Inc., USA). All data underwent one-way analysis of variance (ANOVA) for direct group-to-group comparison, with a *p*-value of <0.05 considered statistically significant. Results are presented as mean ± standard error of the mean (SEM).

### Results and discussion

#### Design

**Fig. 2:**
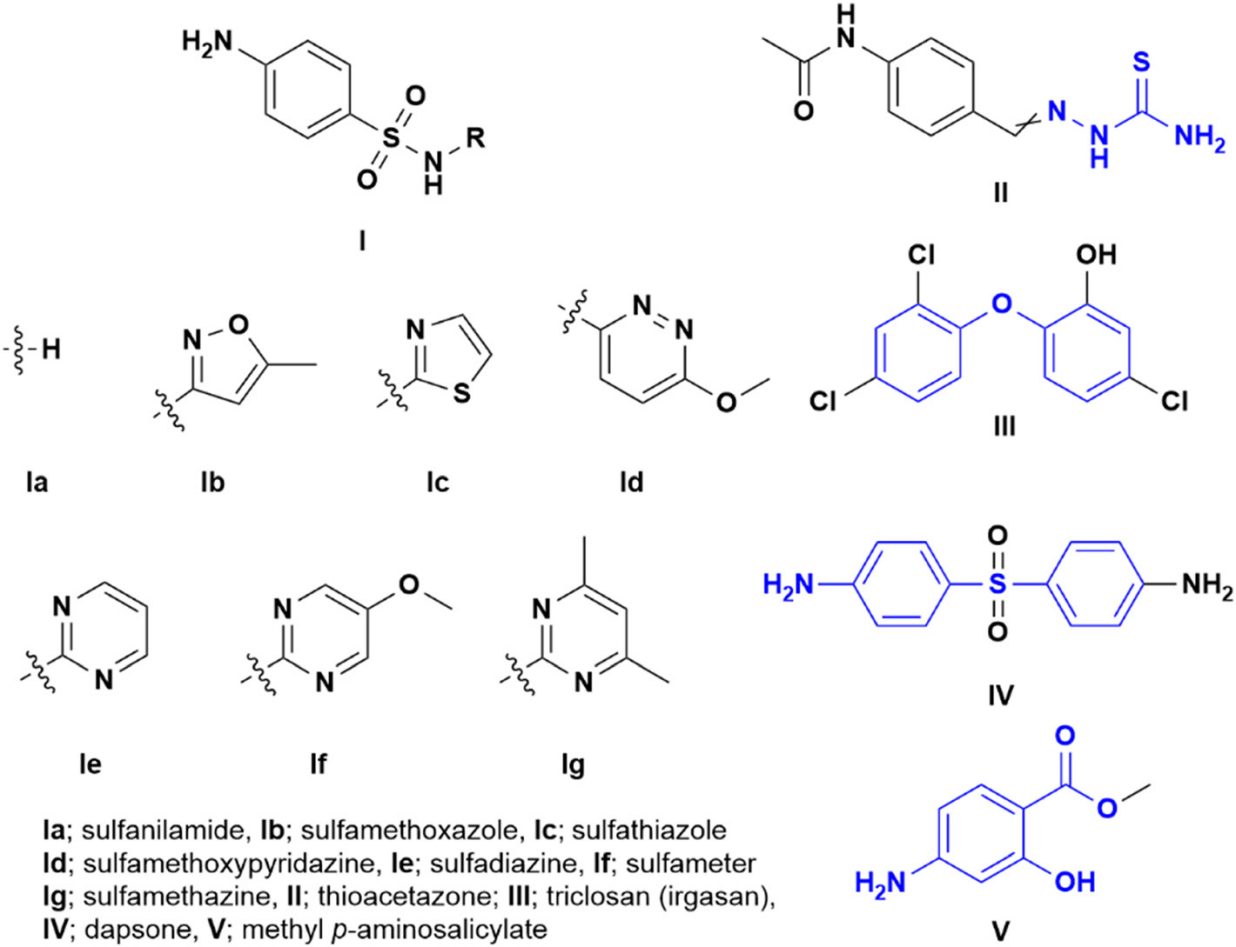
Structure of antimycobacterial drugs used for mutual combination with INH-based acids (sulphonamides **I**, thioacetazone **II**, triclosan **III**, dapsone **IV**, and methyl ester of *p*-aminosalicylic acid **V**).

Initially, we selected various commonly used sulphonamide chemotherapeutics (Fig. 2, **I**) as second partners for hybridization with INH-based hydrazone acid, aiming for dual antimicrobial action. These sulphonamides have demonstrated tuberculostatic activity in numerous studies.^25,26^ By connecting them to INH *via* a hydrolytically labile bond, we aim to harness the combined benefits of both drugs, sulphonamides **Ia**–**Ig** and INH. Thus, the first group (3a–3g) included 4-amino group-linked sulphonamides: sulfanilamide **Ia** (SLF), sulfamethoxazole **Ib** (SMX), sulfathiazole **Ic** (STZ), sulfamethoxypyridazine **Id**, sulfadiazine **Ie** (SDZ), sulfameter **If** (SM), and sulfamethazine 3g (SMZ).

Similarly, we pursued the synthesis of a molecular hybrid of INH with thiosemicarbazide, a vital component of another drug for TB treatment, thioacetazone **II**.^27,28^ Despite the reduced current use of thioacetazone and its associated many drawbacks, it remains valuable in developing countries and for combating drug-resistant TB. Introducing the biologically active thiosemicarbazide fragment into our structure was aimed at potentially reducing its toxicity through modification. To enhance the efficacy of INH, we prepared molecules containing two building blocks of the parent INH. We chose two different approaches: first, direct conjugation of INH with hydrazone acid 2a*via* a hydrazide linkage, and second, linking two INH-hydrazone acids 2a through an amide bond using bifunctional *p*-phenylenediamine. This approach has previously proven to be effective.^29^

Additionally, significant attention was paid to diphenyl ethers. This group forms the basic framework of another well-documented broad-spectrum antimicrobial agent, triclosan (irgasan) **III**, with proven strong antimycobacterial activity.^30,31^ We explored molecular hybrids incorporating several isosteric variants of this scaffold, opting for an amide bond over more hydrolytically labile ester linkage. In some derivatives, chlorine atom(s) present in the original triclosan molecule were introduced.

Other compounds showing antimycobacterial activity, such as the antileprotic drug dapsone^32^**IV** and the antitubercular drug *p*-aminosalicylic acid (PAS,^33,34^ as its methyl ester **V**), were also involved. However, dapsone derivatives are not included in this study due to their unfavourable physicochemical properties (*e.g.*, their low solubility that turned lower after coupling with pyruvic and glyoxylic acids). Instead, we utilized a structurally simpler diphenyl sulphide analogue, anticipating oxidation to the sulfone form *in vivo*.

Also, biphenyl-based compounds have shown a broad spectrum of antimicrobial activity against drug-resistant isolates.^35^

Thus, the second group of hybrids (3h–3k) included combinations of INH 1 with other antimycobacterial scaffolds than sulphonamides: PAS (methyl ester **h**), thiosemicarbazide (**i**), and a second isoniazid molecule linked either directly (3j) or *via* bifunctional *p*-phenylenediamine in the form of acid 2a (3k).

The third group (3l–3q) consisted of amide-linked diphenyl ethers (3l–3o), their thio isostere 3p, which is also an analogue of dapsone, and biphenyl 3q, which represents a structural simplification (direct linkage) altering physical–chemical properties and also stereochemical behaviour.

Lastly, our research also explored variations of previously successful linkers, replacing the pyruvate linker (2a) with a glyoxylic acid-based linker (2b) in several instances.

#### Synthesis

The key intermediates, hydrazone acids 2, were prepared by treating the parent INH with oxocarboxylic acids (pyruvic and glyoxylic acids) in boiling methanol. This method is rapid and highly efficient, yielding over 95% and offering straightforward purification.

**Fig. 3:**
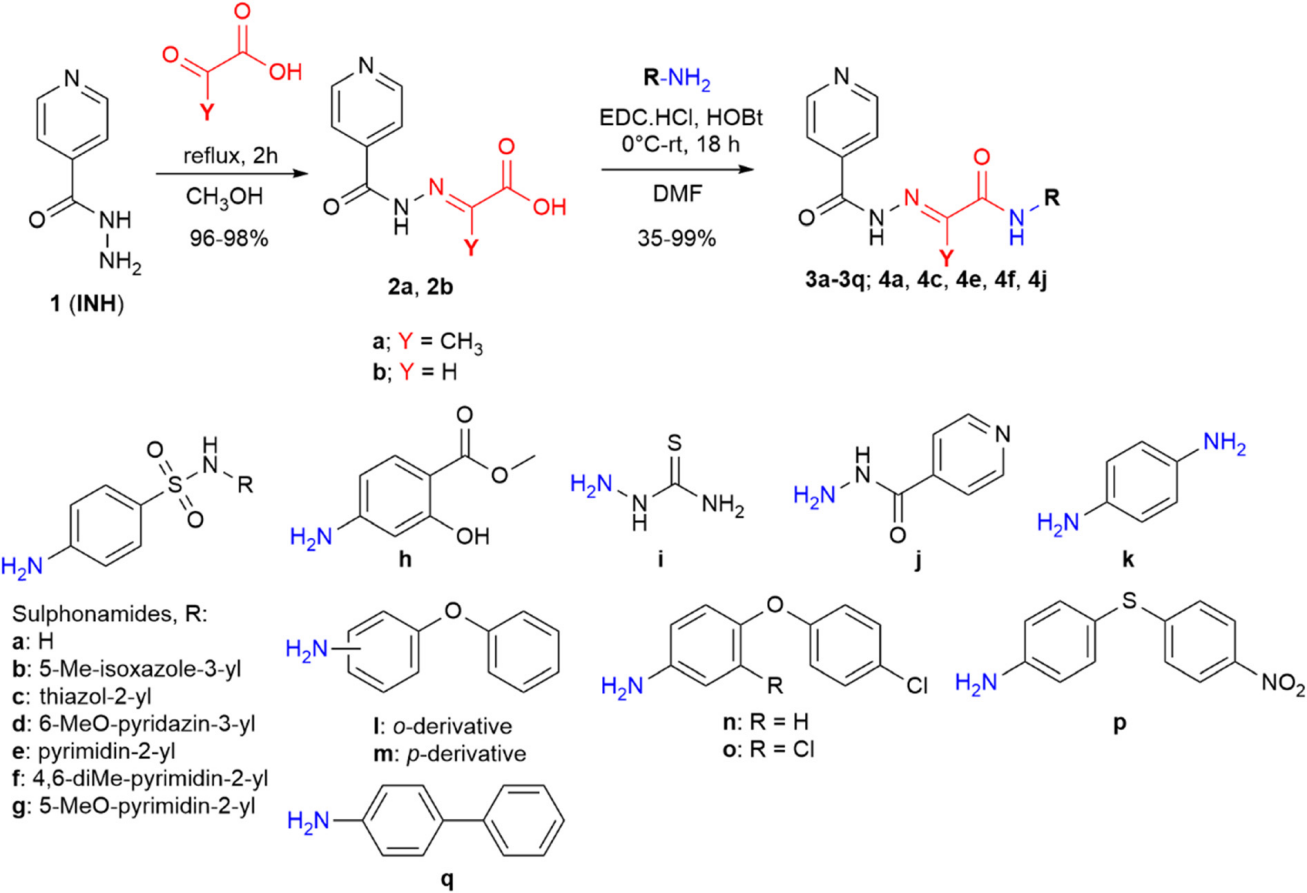
Synthesis of mutual derivatives (DMF: *N*,*N*-dimethylformamide, HOBt: 1-hydroxybenzotriazole, EDC·HCl: 1-ethyl-3-(3-dimethylaminopropyl)carbodiimide hydrochloride).

Due to the specific characteristics of the prepared compounds, it was necessary to consider their synthetic methodology carefully. There are numerous approaches for forming amide bonds; however, the presence of various functional groups in starting materials (and subsequently in the final products) could be incompatible with commonly used reagents. For instance, we aimed to avoid the formation of acyl halogenides of acids 2a and 2b using thionyl chloride, phosphorous chloride, and analogous reagents. Based on our experience, these reactions resulted in an unintended conversion of the pyridine nitrogen atom to its hydrochloride, causing spontaneous crystallization in the solution of the starting material and rendering it unsuitable for further reaction. An even more complex scenario arose upon the subsequent addition of sulphonamide precursors **Ia**–**Ig** to the thus-prepared acyl chlorides. Hence, we opted for the carbodiimide-based coupling method described above as it is among the mildest available for preparing our amides (Fig. 3).

All amides were prepared *via* a carbodiimide-mediated C–N coupling reaction in DMF. HOBt in an equimolar amount was used to promote the reaction, and 1-ethyl-3-(3-dimethylaminopropyl)carbodiimide (EDC) hydrochloride was chosen as the carbodiimide.

This method gave satisfactory reaction conversions (indicated by TLC). However, some reactions provided reduced yields due to a requirement of repeated purification steps, particularly for sulphonamide derivatives 3a–3g derived from acid 2a. Additionally, minor formation of (*Z*)-isomers was observed during the synthesis of these derivatives, which required their separation as impurities. In the case of sulphonamide derivatives obtained from acid 2a, it was essential to use very slow gradient column chromatography for final purification, sometimes repeated. Conversely, derivatives of acid 2b were purified through repeated recrystallization from acetonitrile.

A slight adjustment in the pH of the aqueous solution resulted in a marked increase in water solubility of the sulphonamide derivatives of both investigated acids. During the purification process, this behaviour prevented extraction with any basic or acidic agents, including very diluted ones. However, at least partial purification was achieved through distilled water and brine extraction. Alternatively, while this property is undesirable in synthesising compounds, it becomes desirable for their biological activity, as low water solubility is a well-known drawback of sulphonamide drugs.

This mild extraction was primarily employed to remove the resulting by-product urea from the coupling reagent and HOBt, both highly soluble in water. While simple extraction alone could not purify crude products to an acceptable level in most cases, it served as a preliminary purification step before crystallization or preparative chromatography. On the other hand, the derivative 3k incorporating a phenylene diamine linker was prepared with a nearly quantitative yield (99%). It required no further purification, as it became practically insoluble in the reaction solvent used.

Overall yields for other derivatives ranged widely, with sulphonamide derivatives achieving a minimum yield of 35% (3e) and the phenylene diamine derivative 3k achieving a yield of 99%.

### Antimicrobial activity

#### Antimycobacterial activity

As a primary goal, we conducted *in vitro* screening of all INH-based compounds 2–4 against three mycobacterial strains. These included fully drug-susceptible *Mycobacterium tuberculosis* H37Rv strain 331/88, polydrug-resistant *Mycobacterium avium* 330/88, and a clinical isolate of *M. kansasii* 6509/96. The parent isoniazid 1 served as a reference compound for comparison of minimum inhibitory concentrations (MICs), detailed in Table 1. Notably, almost all derivatives share significant antimycobacterial action.

**Table 1:** Antimycobacterial activity of the INH-based compounds 2–4

MIC [μM]								
Code	R	*Mtb* 331/88		*M. avium* 330/88		*M. kansasii* 6509/96		
		14 d	21 d	14 d	21 d	7 d	14 d	21 d
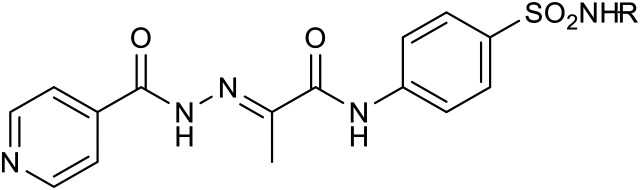								
3a	H	2	2	≥1000	≥1000	32	64	64
3b	5-Me-isoxazole-3-yl	**0.5**	1	1000	≥1000	16	32	32
3c	Thiazol-2-yl	1	1	≥1000	≥1000	16	16	16
3d	6-MeO-pyridazin-3-yl	1	1	≥1000	≥1000	32	32	32
3e	Pyrimidin-2-yl	2	2	1000	≥1000	32	64	64
3f	4,6-DiMe-pyrimidin-2-yl	1	1	500^a^	500^a^	16	32	32
3g	5-MeO-pyrimidin-2-yl	2	2	≥1000	≥1000	32	32	32
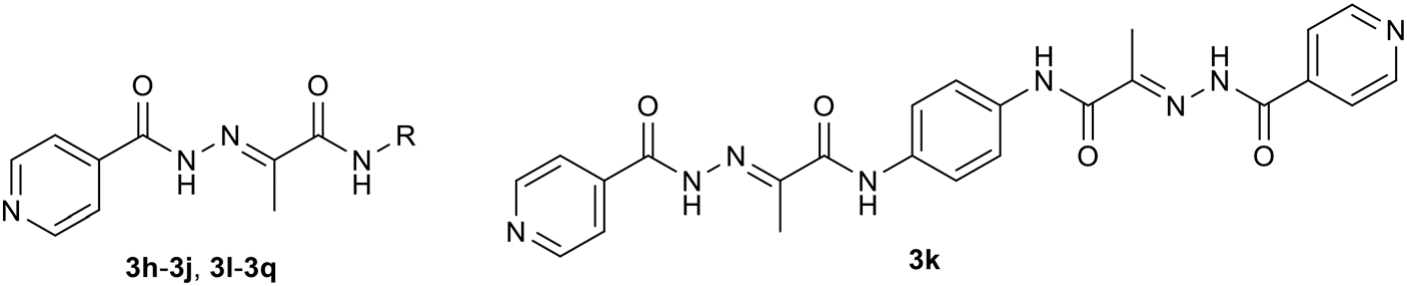								
3h	3-OH-4-(MeOCO)-phenyl	**0.5**	**0.5**	≥1000	≥1000	8	16	16
3i	NH_2_CSNH-	1	2	1000	1000	16	32	32
3j	Isonicotinamido	**0.25**	**0.5**	500	500	8	16	16
3k	—	64^a^	64^a^	250^a^	250^a^	250^a^	250^a^	250^a^
3l	2-PhO-phenyl	**0.25**	**0.5**	≥1000	≥1000	4	8	16
3m	4-PhO-phenyl	**0.25**	**0.25**	500	500	**≤1**	**≤1**	**2**
3n	4-(4-Cl-PhO)phenyl	**0.5**	**0.5**	500^a^	500^a^	**1**	**2**	4
3o	3-Cl-4-(4-Cl-PhO)phenyl	**0.5**	**0.5**	500^a^	500^a^	32	64	64
3p	4-[(4-NO_2_-phenyl)thio]phenyl	**0.25**	**0.25**	500^a^	500^a^	**1**	**1**	**2**
3q	Biphenyl-4-yl	**≤0.25**	**≤0.25**	>250	>250	8	8	16
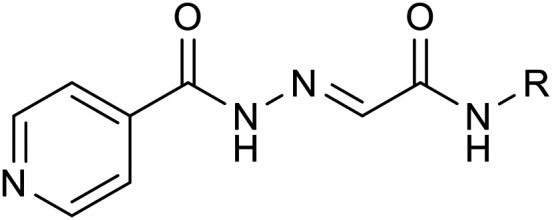								
4a	H	1	2	1000	≥1000	16	32	32
4c	Thiazol-2-yl	2	4	**250**	500	8	8	8
4e	Pyrimidin-2-yl	1	2	500	500	8	16	16
4f	4,6-DiMe-pyrimidin-2-yl	1	2	1000	≥1000	8	16	16
4j	Isonicotinamido	1	2	1000	>1000	8	16	16
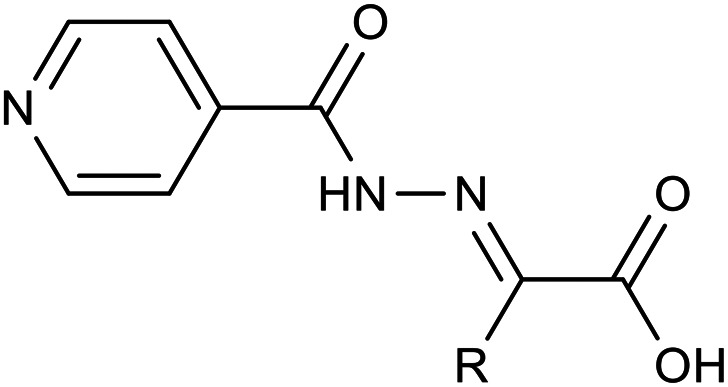								
2a	CH_3_	1	1	500	>1000	8	16	16
2b	H	**0.25**	**0.5**	**250**	>250	4	4	8
1, INH	—	1	1	>250	>250	8	8	16

a At this concentration, the growth of the pathogen was observed; however, at the next higher concentration, turbidity was present, preventing the exact determination of the MIC value.

Initially, we assessed the activity of two INH-based acids: the pyruvic acid derivative 2a and the glyoxylic acid-based derivative 2b. The first one maintained efficacy comparable to its INH precursor, while the latter hydrazone acid exhibited slightly higher effectiveness across all mycobacterial strains (up to 4× for *Mtb*).

Encouraged by these findings, we proceeded to synthesize their amides, starting with seventeen compounds derived from acid 2a and subsequently five (predominantly sulphonamide-based) derivatives from acid 2b.

The first group included 4-amino group-linked sulphonamides (3a–3g). These dual compounds displayed consistent activity among them. Their MIC values against *Mtb* ranged from 0.5 to 2 μM, with the SMX derivative 3b being the most potent (0.5–1 μM). In contrast, the SLF 3a, SDZ 3e, and SM 3g derivatives were somewhat less effective (MIC = 2 μM), all comparable to the standard INH. The efficacy against *M. avium* was low (MIC starting from 1000 μM), whereas *M. kansasii* showed a moderate susceptibility with MIC values ranging from 16 to 64 μM, favouring the STZ derivative 3c (16 μM). Again, the SLF and SM derivatives were slightly less effective. The activity compared to the parent INH was mildly reduced against this strain.

The second group included combinations of INH 1 with other antimycobacterial scaffolds than sulphonamides. Except for the inactive compound 3k with a *p*-phenylenediamine linker, these modifications were more successful than the sulphonamides. “Doubling” INH in a single molecular entity increased activity against *Mtb* up to a MIC value of 0.25 μM, and attaching the methyl ester of PAS led to a MIC of 0.5 μM. The thioacetazone analogue was as active as INH, based on a comparison of MIC values. Activity against *M. avium* started at 500 μM, and the results were comparable to the parent compound 1 for *M. kansasii*.

The third group, generally the most effective one, consisted of amide-linked diphenyl (thio)ethers (3l–3p) and biphenylamide 3q. All derivatives displayed exceptional activity against *Mtb* with MIC values of ≤0.25–0.5 μM. Non-halogenated 4-yl derivatives (3m, 3p, and 3q), mainly the biphenyl analogue 3q (≤0.25 μM), were the most effective. Generally, halogenation, resulting in increased lipophilicity, did not lead to enhanced activity but only maintained it compared to non-chlorinated compounds. However, these modifications did not result in compounds inhibiting *M. avium* at low concentrations (MIC ≥500 μM). In contrast, this series successfully obtained derivatives with excellent activity against *M. kansasii*, as two compounds (3l and 3q) were fully comparable to INH, and three of them showed increased activity (MIC of ≤1–4 μM). In all cases, these are 4-phenoxyaniline derivatives (3m and 3n) or the thioether analogue 3p. Here, introducing one chlorine leads only to a slightly lower activity (3m*vs.*3n). In contrast, two chlorine atoms have a clearly unfavourable effect (3m*vs.*3o, with an increase in MIC up to 64 times). In contrast to *Mtb*, excellent activity against *M. kansasii* requires the presence of a bridge connecting two benzene rings (3m*vs.*3q).

In summary, the compounds prepared (3a–3q) maintained or enhanced the efficacy compared to the parent INH 1 against *Mtb* and *M. kansasii*. However, their activity against *M. avium* remained negligible and one or several orders of magnitude lower.

Since the hydrazone acid derived from glyoxylic acid 2b proved to be more effective than that prepared from pyruvic acid (2a), we also prepared its several sulphonamide derivatives (4a, 4c, 4e, and 4f) and a “double” INH derivative 4j. However, this modification was unsuccessful, as the MIC values for *Mtb* obtained (1–4 μM) were not better than those for the amides 3 and lower than those for the parent acid 2b. Contrarily, although still not impressive, a slightly higher *in vitro* efficacy was identified for *M. avium* (250 μM for the STZ derivative 4c and 500 μM for the SDZ analogue 4e). There was also a slight increase in activity against *M. kansasii*, with MIC values of 8–32 μM. Again, the STZ derivative 4c (8 μM) was the most effective, while the most hydrophilic SLF derivative 4a was the least effective (16–32 μM).

Having these results in hand, to confirm our design and hypothesis, we assessed the antimycobacterial activity of the parent small molecules used to synthesize the most *in vitro* active amides (3h, 3l–3q; Table 2). The activities of sulphonamides were published previously by our group.^26^ They exhibited activity against both *Mtb* (MIC 32–125 μM, with SMX being the most effective) and NTM (MIC 1–125 μM, with a superiority of STZ). In general, sulphonamides, particularly, SMX and STZ, were significantly more effective than the other precursors used in this study, except for INH and partially the polyhalogenated aniline **o** and the bacteriostatic nitro precursor **p**.

**Table 2:** Antimycobacterial activity of precursors

MIC [μM]								
Precursor	Name	*Mtb* 331/88		*M. avium* 330/88		*M. kansasii* 6509/96		
		14 d	21 d	14 d	21 d	7 d	14 d	21 d
**h**	Methyl *p*-aminosalicylate	250	250	>1000	>1000	1000	>1000	>1000
**l**	2-Phenoxyaniline	250	250	500	1000	500	500	500
**m**	4-Phenoxyaniline	500	500	1000	1000	250	500	500
**n**	4-(4-Cl-phenoxy)aniline	250	500	1000	1000	250	500	500
**o**	3-Cl-4-(4-Cl-phenoxy)aniline	**125**	**125**	**125**	**125**	**125**	**125**	**125**
**p**	4-[(4-Nitrophenyl)thio]aniline	**125**	≥1000	≥1000	≥1000	**8**	≥1000	≥1000
**q**	Biphenyl-4-amine	500	500	≥1000	≥1000	250	250	500

The activities of the majority of the amines ranged from 250 to ≥1000 μM, with two notable exceptions (in addition to sulphonamides): the lipophilic 3-chloro-4-(4-chlorophenoxy)aniline **o**, which consistently exhibited MIC values of 125 μM, and 4-[(4-nitrophenyl)thio]aniline **p**, an analogue of antileprotic drug dapsone, which demonstrated significant inhibition of *Mtb* after two weeks of incubation and especially of *M. kansasii* after seven days of incubation. After an extended time of incubation, the activity diminished, indicating bacteriostatic rather than bactericidal action. Compared to INH, these values are generally lower, but their combination with it into a single molecular entity resulted in a substantial enhancement of activity. This increase may be attributed to several factors: 1) compared to the parent INH, the enhanced lipophilicity may facilitate passive diffusion through the highly lipophilic mycobacterial cell wall; 2) the potential additive or synergistic effects of the amines and INH; 3) the influence on its metabolism. To highlight the significance, combining two pharmacophores within a single mutual molecule provides lower MIC values than either component alone, emphasising the potential of such a combination.

Four potent amides that demonstrated the highest *in vitro* efficacy against drug-susceptible *Mtb* strain 331/88 (specifically, the most active sulphonamide SMX-derivative 3b, the most active diaryl ether 3m, the dapsone analogue 3p, and the biphenyl derivative 3q) and were further tested against a panel of six multidrug-resistant TB strains with different resistance profiles (Table 3). Notably, one of these isolates (Praha 131) met previous criteria for extensively drug-resistant TB, showing resistance to INH, RIF, any fluoroquinolone, and at least one of three injectable agents—kanamycin, amikacin, and capreomycin (here, namely ofloxacin and amikacin).

**Table 3:** Activity against MDR-TB strains

Code	MIC [μM]													
	*Mtb* Praha 1		*Mtb* Praha 4		*Mtb* Praha 131		*Mtb* 234/2005		*Mtb* 9449/2007		*Mtb* 7357/1998		*Mtb* 8666/2010	
	14 d	21 d	14 d	21 d	14 d	21 d	14 d	21 d	14 d	21 d	14 d	21 d	14 d	21 d
3b	NT	NT	NT	NT	≥250	≥250	NT	NT	≥250	≥250	NT	NT	≥250	≥250
3m	**16**	**16**	**16**	**16**	**16**	**16**	**8**	**16**	**16**	**16**	**16**	**16**	**16**	**16**
3p	NT	NT	NT	NT	≥250	≥250	NT	NT	≥250	≥250	NT	NT	≥250	≥250
3q	32	64	64	64	32	64	64	125	64	64	64	125	64	64
OFX	1.4	1.4	>22	>22	22	22	0.7	0.7	2.8	2.8	11	11	11	11
GEN	1	1	0.5	0.5	>17	>17	0.26	0.26	1	1	1	1	4	4

Here, two compounds (the SMX and nitro derivatives 3b and 3p, respectively) exhibited only negligible activity with MIC values of ≥250 μM, indicating a complete cross-resistance to INH that the presence of a second bioactive antimycobacterial molecule could not overcome. On the other hand, the diphenyl ether and biphenyl derivatives (3m, 3q) retained their antimycobacterial activity, although at significantly higher concentrations starting from 8 μM, with the diphenylether-4-yl 3m showing the best activity in the range of 8–16 μM. The efficacy of these compounds remained generally consistent across different resistance profiles. These results highlighted the significance of the presence of two benzene rings, either directly linked or connected *via* an oxygen atom in the amidic part of these mutual molecules 3, in maintaining antimycobacterial efficacy.

We designed compounds with a dual mechanism of action, where the highly potent effect of INH would be complemented by another antimicrobial molecule targeting a distinct biological pathway. For example, sulphonamides and PAS target folate metabolism, and nitro compounds generate reactive nitrogen species, while diphenyl ethers offer an alternative type of InhA inhibition directly without requiring bioactivation; a deficiency in bioactivation is the most frequent cause of INH resistance. The goal was to ensure that these mechanisms would function in a synergistic manner or be indifferent to each other while maintaining efficacy in the presence of INH resistance. The complementarity of these mechanisms, fundamentally different or supplementary to InhA inhibition, would ideally address resistance by affecting both pathways.

Based on these results and by analogy with previous studies,^13,14^ it can be concluded that the predominant mechanism of action is InhA inhibition, similar to that of INH. However, the partial efficacy observed for some derivatives (mainly 3m) against INH-resistant strains, and even significantly lower MIC values than what was found for INH itself, can be attributed to a dual mechanism of action, where the secondary effect is less potent compared to primary InhA inhibition.

Regarding our design idea, both scenarios for action of these derivatives are plausible: (1) hydrolysis into the parent compounds (*i.e.*, two active antimycobacterial active agents and a by-product such as pyruvic or glyoxylic acid), behaving as mutual prodrugs, or (2) functioning as intact molecular hybrids without any need for hydrolysis.

#### Antibacterial and antifungal activity

Although the tested compounds were designed primarily as potential antimycobacterial agents, we expanded our testing to include evaluation against eight additional bacterial strains (both Gram-positive and Gram-negative) and eight fungal strains (yeasts and moulds). This comprehensive approach provides a thorough overview of their activity profile, considering that some small molecules used for hybridization harbour additional antimicrobial molecules (mainly sulphonamides and diphenyl ethers).

Based on the findings, none of the compounds demonstrated antifungal activity, as all exhibited MIC values above 500 μM against all tested fungal strains. Regarding antibacterial activity, it was generally weak. Only three compounds showed an inhibitory effect against Gram-positive strains but at higher concentrations starting from 125 μM. Namely, thiosemicarbazide 3i inhibited *S. epidermidis* at 125 μM, while the *p*-aminosalicylic derivative 3h showed identical activity against the same strain at the same concentration. “Bis”-INH derivative 3j showed MIC values of 250 μM and 500 μM against *S. epidermidis* and MRSA strains, respectively. These results highlight the compounds' selective antimycobacterial activity, emphasizing their potential for targeting *Mycobacterium tuberculosis* and NTM while demonstrating limited effectivity against other microbial species.

#### Haemolytic activity *ex vivo*

Human red blood cells (hRBCs) were utilized as a biological model to assess cytotoxicity and biocompatibility (haemocompatibility) with the human biological system. Five approved antitubercular drugs, namely bedaquiline, RIF, INH, EMB, and pyrazinamide, along with three selected newly synthesized highly active derivatives (3m, 3p, and 3q), were included in the *ex vivo* hRBC haemolysis assay.

**Fig. 4:**
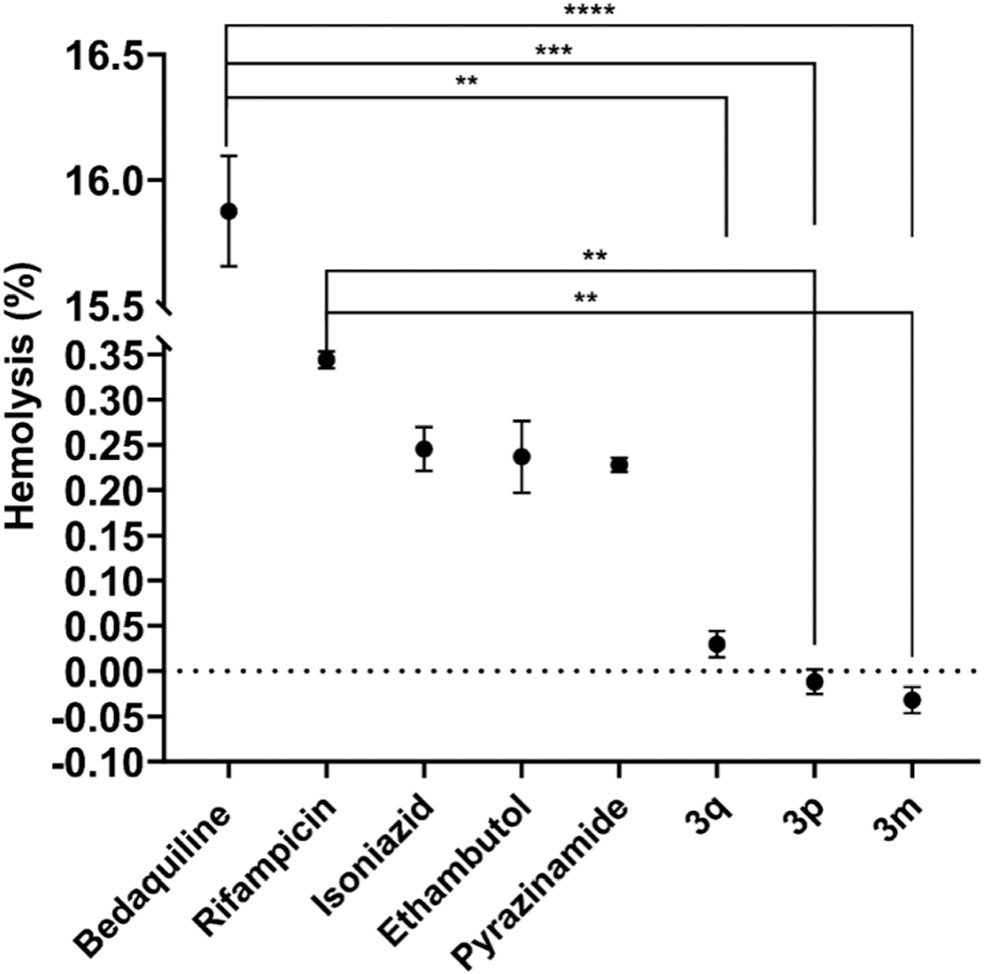
Comparison of haemolytic activity between approved antituberculosis drugs (bedaquiline, RIF, INH, EMB, and pyrazinamide) and derivatives 3q, 3p, and 3m was conducted using an *ex vivo* human red blood cell (hRBC) haemolysis assay. The assay involved exposing hRBCs to the compounds for 1 hour at 37 °C, and then measuring released haemoglobin in the supernatant at 405 nm. Data were normalized against a negative control (unexposed hRBCs) and provided by hexaplicates ± SEM. Statistical significance was assessed using nonparametric one-way ANOVA (Kruskal–Wallis' test) by *p*-values, with significance at *p* < 0.05.

As depicted in Fig. 4, only a negligible haemolytic activity (haemolysis rate from 0.01% to 0.35%) was observed at final concentrations equivalent to 125 μM of the included established drugs and one candidate derivative (RIF, INH, EMB, pyrazinamide, and biphenyl-based amide 3q). In contrast, the results indicated that bedaquiline adversely affected the lipid bilayer of hRBC membranes, resulting in significant haemolysis. This adverse effect on mammalian red blood cells (rat erythrocytes) was also demonstrated in the study of Belosludtsev *et al.*^36^ Notably, derivatives 3m and 3p exhibited even a slight membrane-stabilizing effect on hRBC, while registered haemolysis was slightly lower than in drug-unexposed erythrocytes (negative control). Statistical analysis revealed that when compared to RIF, these two derivatives exhibited higher biocompatibility with the human biological system. Furthermore, compared to bedaquiline, all three derivatives (3m, 3p, 3q) demonstrated better tolerability.

## Conclusions

This study is focused on the synthesis and evaluation of mutual bioactive amides, which combine INH-based hydrazone acids with antimicrobial active compounds sharing the NH_2_ group (sulphonamides, a second INH molecule, PAS, thiosemicarbazide, biphenyl, and diphenyl (thio)ethers). The objective was to improve antimycobacterial activity through a dual-action approach.

The synthesized compounds exhibited excellent activity against *Mtb*. Many compounds were more effective or at least comparable to the parent INH, particularly diphenyl (thio)ether and biphenyl analogues—similar conclusions, although with higher nominal MIC values, were found for *M. kansasii*. The inhibition of *M. avium* was significantly lower, with the best results being modest and not clinically compelling. Several meaningful structure–activity relationships were identified.

A noteworthy finding was that two compounds showed significant *in vitro* efficacy against six MDR-TB strains, although with higher MIC values compared to the drug-susceptible *Mtb* strain. This activity highlights the importance of continued optimization of INH-based derivatives to provide even better activity against resistant TB strains.

Additionally, the antimicrobial spectrum of the novel derivatives extended beyond mycobacteria, with some compounds showing mild inhibitory effects observed against Gram-positive bacteria. However, this activity was generally minimal, along with no inhibition of human pathogenic fungi, indicating a selectivity for mycobacteria. This feature was also confirmed by toxicity evaluation. The three most active compounds were well-tolerated at a supra-MIC concentration of 125 μM without significant haemolysis, suggesting a broad therapeutic window. This also highlights that even a relatively simple chemical modification of the parent INH can reduce toxicity without compromising efficacy.

In summary, all these findings suggest potential pathways for further modifications of INH-based compounds to enhance their efficacy and expand the spectrum of activity, continuing the development of potential drugs for the effective treatment of TB, particularly drug-resistant strains.

## Data availability

The data supporting this article have been included as part of the published article and its ESI.†

## Author contributions

M. K. was responsible for conceptualization; V. P., J. S., K. K., and J. Ő. were responsible for the methodology; V. P., J. S., K. K., J. Ő., O. J., and M. K. were responsible for investigation; V. P., K. K., and M. K. were responsible for writing – original draft preparation; V. P., K. K., J. Ő., O. J., and M. K. were responsible for writing – review and editing; K. K. and M. K. were responsible for supervision; M. K. was responsible for funding acquisition.

## Conflicts of interest

There are no conflicts to declare.

## Supplementary Material

MD-016-D4MD00663A-s001
