# Supplementary material for: Enhancing the antimycobacterial efficacy of pyridine-4-carbohydrazide: linkage to additional antimicrobial agents via oxocarboxylic acids
Source: RSC Med Chem. 2024 Oct 16;16(2):767–78. doi: 10.1039/d4md00663a (PMC11575622; doi:10.1039/d4md00663a)

Supplementary Information (SI) for RSC Medicinal Chemistry.
This journal is © The Royal Society of Chemistry 2024

Enhancing the Antimycobacterial Efficacy of Pyridine-4-Carbohydrazide: Linkage to Additional Antimicrobial Agents via Oxocarboxylic Acids

Václav Pflégr [a], Klára Konečná [b], Jiřina Stolaříková [c], Jan Ősterreicher [b], Ondřej Janďourek [b] and Martin Krátký [a,*]

[a] Department of Organic and Bioorganic Chemistry, Faculty of Pharmacy in Hradec Králové, Charles University, Akademika Heyrovského 1203, 500 03 Hradec Králové, Czech Republic
[b] Department of Biological and Medical Sciences, Faculty of Pharmacy in Hradec Králové, Charles University, Akademika Heyrovského 1203, 500 03 Hradec Králové, Czech Republic
[c] Laboratory for Mycobacterial Diagnostics and Tuberculosis, Regional Institute of Public Health in Ostrava, Partyzánské náměstí 7, Ostrava, Czech Republic
* Corresponding author: martin.kratky@faf.cuni.cz; Tel.: +420-495067302; Fax: +420-495067166

1. Characterization of the prepared compounds

(*E*)-2-(2-Isonicotinoylhydrazineylidene)-*N*-(4-sulfamoylphenyl)propanamide **3a**

^{1}H NMR (500 MHz, DMSO-d_6) δ 11.13 (1H, s, CONH-N), 10.62 (1H, s, NH-Ph), 8.58 – 8.53 (2H, s, H2, H6), 8.00 – 7.92 (4H, m, H2′, H3′, H5′, H6′), 7.78 – 7.71 (2H, m, H3, H5), 7.23 (2H, s, NH_2), 2.13 (3H, s, CH_3). ^{13}C NMR (126 MHz, DMSO) δ 167.74, 165.59, 149.37, 148.06, 142.53, 137.67, 126.69, 122.52, 118.85, 11.32. IR (ATR-Ge): 657, 702, 737, 753, 813, 834, 930, 997, 1034, 1102, 1156, 1187, 1259, 1293, 1322, 1402, 1453, 1490, 1515, 1593, 1612, 1668, 1685, 1923, 2539, 3217 cm^{-1}. Elemental analysis $C_{15}H_{15}N_5O_4S$ (361.38); calculated C, 49.86; H, 4.18; N, 19.38, found C, 49.99; H, 4.01; N, 19.33. Beige solid; yield 88%, mp: 277-279°C. R_f 0.3.

(*E*)-2-(2-Isonicotinoylhydrazineylidene)-*N*-{4-[*N*-(5-methylisoxazol-3-yl)sulfamoyl]phenyl}-propanamide **3b**

^{1}H NMR (500 MHz, DMSO-d_6) δ 11.40 – 11.21 (2H, m, CONH-N, SO_2NH-), 10.44 (1H, s, NH-Ph), 8.78 (2H, d, J = 5.0 Hz, H2, H6), 7.94 (2H, s, H3, H5), 7.85 – 7.77 (4H, m, H2′, H3′, H5′, H6′), 6.13 (1H s, CH_{Iso}), 2.30 (3H, s, $CH_{3\,Iso}$), 2.22 (3H, s, CH_3). ^{13}C NMR (126 MHz, DMSO) δ 170.45, 163.55, 157.75, 150.27, 149.7, 142.64, 140.81, 140.80, 133.94, 128.08, 122.25, 120.10, 95.56, 12.22, 10.0. IR (ATR-Ge): 729, 837, 957, 1005, 1022, 1084, 1129, 1155, 1183, 1223, 1271, 1303, 1374, 1405, 1419, 1473, 1530, 1589, 1650, 1674, 3264 cm^{-1}. Elemental analysis $C_{19}H_{18}N_6O_5S$ (442.45); calculated C, 51.58; H, 4.10; N, 18.99, found C, 51.69; H, 4.00; N, 18.52. Grey solid; yield 47%, mp: 221-222°C. R_f 0.4.

(*E*)-2-(2-Isonicotinoylhydrazineylidene)-*N*-{4-[*N*-(thiazol-2-yl)sulfamoyl]phenyl}propanamide **3c**

^{1}H NMR (500 MHz, DMSO-d_6) δ 12.70 (1H, s, SO_2NH-), 11.29 (1H, s, CONH-N), 10.36 (1H, s, NH-Ph), 8.78 (2H, d, J = 5.1 Hz, H2, H6), 7.89 (2H, s, H3, H5), 7.77 – 7.73 (4H, m, H2′, H3′, H5′, H6′), 7.25 (1H, dd, J = 4.6, 0.8 Hz, CH_{Thz}), 6.82 (dd, J = 4.6, 0.8 Hz, CH_{Thz}), 2.22 (3H, s, CH_3). ^{13}C NMR (126 MHz, DMSO) δ 168.89, 163.26, 150.95, 150.29, 141.52, 140.75, 137.17, 127.00, 124.60, 122.28, 119.85, 108.31, 55.08, 12.58. IR (ATR-Ge): 612, 630, 659, 689, 720, 834, 859, 930, 1004, 1089, 1140, 1182, 1270, 1315, 1375, 1405, 1429, 1519, 1570, 590, 1682, 3241 cm^{-1}. Elemental analysis $C_{18}H_{16}N_6O_4S_2$ (444.48); calculated C, 48.64; H, 3.63; N, 18.91, found C, 48.85; H, 3.77; N, 19.05. Yellow solid; yield 35%, mp: 248-249°C. R_f 0.3.

(*E*)-2-(2-Isonicotinoylhydrazineylidene)-*N*-{4-[*N*-(6-methoxypyridazine-3-yl)sulfamoyl]phenyl}-propanamide **3d**

^{1}H NMR (500 MHz, DMSO-d_6) δ 12.97 (1H, s, SO_2NH-), 11.35 (1H, s, CONH-N), 10.39 (1H, s, NH-Ph), 8.77 (2H, d, J = 4.7 Hz, H2, H6), 8.07 – 7.54 (7H, m, H3, H5, H2′, H3′, H5′, H6′, $H4_{Pyridaz}$), 7.37 (1H, J = 9.8 Hz, $H5_{Pyridaz.}$), 3.84 (3H, s, OCH_3), 2.22 (3H, s, CH_3). ^{13}C NMR (126 MHz, DMSO) δ 167.52, 163.39, 155.30, 151.00, 149.29, 141.49, 140.15, 136.00, 127.37, 122.27, 121.04, 119.88, 115.02, 54.67, 12.59. IR (ATR-Ge): 670, 701, 729, 834, 957, 1005, 1023, 1128, 1156, 1182, 1222, 1271, 1372, 1406, 1419, 1473, 1530, 1588, 1650, 1673, 2950, 3069, 3249 cm^{-1}. Elemental analysis $C_{20}H_{19}N_7O_5S$ (469.48); calculated C, 51.17; H, 4.08; N, 20.88, found C, 51.00; H, 4.32; N, 21.00. Pale yellow solid; yield 40%, mp: 213-215°C. R_f 0.4.

(*E*)-2-(2-Isonicotinoylhydrazineylidene)-*N*-{4-[*N*-(pyrimidine-2-yl)sulfamoyl]phenyl}propanamide **3e**

^{1}H NMR (500 MHz, DMSO-d_6) δ 11.75 (1H, s, SO_2NH-), 11.30 (1H, s, CONH-N), 10.44 (1H, s, NH-Ph), 8.78 (2H, s, H2, H6), 8.50 (2H, d, J = 4.8 Hz, H2, H6 $_{Pyrimid.}$), 7.96 – 7.92 (4H, m, H3, H5, H3′, H5′), 7.79 (2H, s, H2′, H6′), 7.04 (1H, t, J = 4.8 Hz, H4 $_{Pyrimid.}$), 2.22 (3H, s, CH_3). ^{13}C NMR (126 MHz, DMSO) δ 163.56, 158.57, 157.12, 151.15, 150.34, 142.33, 140.50, 134.88, 129.82,

128.93, 122.28, 119.68, 116.00, 12.60. IR (ATR-Ge): 661, 692, 799, 836, 952, 1094, 1152, 1155, 1186, 1362, 1378, 1409, 1423, 1435, 1447, 1508, 1515, 1542, 1583, 1555, 1647, 1697, 3255 cm^{-1}. Elemental analysis $C_{19}H_{17}N_7O_4S$ (439.45); calculated C, 51.93; H, 3.90; N, 22.31, found C, 52.15; H, 3.99; N, 22.11. Beige solid; yield 35%, mp: 253-255°C. R_f 0.4.

(*E*)-*N*-{4-[*N*-(4,6-Dimethylpyrimidine-2-yl)sulfamoyl]phenyl}-2-(2-isonicotinoylhydrazine-ylidene)propanamide **3f**
^{1}H NMR (500 MHz, DMSO-d_6) δ 11.64 (1H, s, SO_2NH-), 11.29 (1H, s, CONH-N), 10.35 (1H, s, NH-Ph), 8.78 (2H, d, J = 4.9 Hz, H2, H6), 8.09 – 7.64 (6H, m, H3, H5, H2′, H6′, H3′, H5′), 6.75 (1H, s, H4 $_{Pyrimid.}$), 2.27 – 2.20 (9H, m, 2 × CH_3 $_{Pyrimid.}$, CH_3 $_{linker}$). ^{13}C NMR (126 MHz, DMSO) δ 167.54, 163.39, 156.36, 151.15, 150.28, 141.94, 140.74, 135.35, 129.33, 122.28, 119.23, 113.68, 112.00, 23.03, 12.50. IR (ATR-Ge): 656, 683, 708, 727, 786, 833, 871, 976, 1007, 1082, 1093, 1153, 1182, 1214, 1248, 1298, 1329, 1368, 1409, 1429, 1514, 1590, 1668, 1712, 3296 cm^{-1}. Elemental analysis $C_{21}H_{21}N_7O_4S$ (467.50); calculated C, 53.95; H, 4.53; N, 20.97, found C, 54.12; H, 4.68; N, 20.72. White solid; yield 61%, mp: 291-292°C. R_f 0.4.

(*E*)-2-(2-Isonicotinoylhydrazineylidene)-*N*-{4-[*N*-(5-methoxypyrimidine-2-yl)sulfamoyl]phenyl}-propanamide **3g**
^{1}H NMR (500 MHz, DMSO-d_6) δ 11.43 (1H, s, SO_2NH-), 11.30 (1H, s, CONH-N), 10.43 (1H, s, NH-Ph), 8.78 (2H, d, J = 4.4 Hz, H2, H6), 8.29 (2H, s, H2, H6 $_{Pyrimid.}$), 7.95 – 7.90 (4H, m, H3, H5, H3′, H5′), 7.79 (2H, s, H2′, H6′), 3.79 (3H, s, OCH_3), 2.22 (3H, s, CH_3). ^{13}C NMR (126 MHz, DMSO) δ 163.56, 151.15, 150.35, 149.83, 144.85, 142.22, 135.09, 129.82, 128.78, 122.27, 119.72, 112.35, 56.45, 12.61. IR (ATR-Ge): 614, 685, 712, 761, 785, 836, 859, 929, 955, 1014, 1093, 1153, 1183, 1246, 1276, 1296, 1336, 1403, 1426, 1450, 1494, 1514, 1587, 1645, 1687, 3236 cm^{-1}. Elemental analysis $C_{20}H_{19}N_7O_5S$ (469.48); calculated C, 51.17; H, 4.08; N, 20.88, found C, 51.11; H, 4.19; N, 20.97. Beige solid; yield 52%, mp: 186-187°C. R_f 0.5.

Methyl (*E*)-2-hydroxy-4-[2-(2-isonicotinoylhydrazineylidene)propanamido]benzoate **3h**
^{1}H NMR (500 MHz, DMSO-d_6) δ 11.30 (1H, s, CONH-N), 10.62 (1H, s, OH), 10.32 (1H, s, NH-Ph), 8.78 (2H, s, H2, H6), 7.81 – 7.73 (3H, m, H3, H5, H6′) 7.60 – 7.45 (1H, m, H3′), 7.36 – 7.25 (1H, m, H5′), 3.87 (3H, s, OCH_3), 2.22 (3H, s, CH_3). ^{13}C NMR (126 MHz, DMSO) δ 169.42, 163.83, 161.51, 151.34, 150.55, 145.08, 144.45, 141.00, 131.21, 122.58, 111.73, 108.49, 107.47, 52.73, 12.87. IR (ATR-Ge): 640, 671, 698, 737, 765, 791, 840, 870, 929, 960, 983,1003, 1067, 1103, 1146, 1162, 1191, 1232, 1247, 1273, 1293, 1350, 1370, 1415, 1444, 1528, 1554, 1564, 1617, 1673, 3287 cm^{-1}. Elemental analysis $C_{17}H_{16}N_4O_5$ (356.34); calculated C, 57.30; H, 4.53; N, 15.72, found C, 57.52; H, 4.71; N, 15.97. Yellow solid; yield 94%, mp: 214-215°C. R_f 0.6.

(*E*)-2-[2-(2-Isonicotinoylhydrazineylidene)propanoyl]hydrazine-1-carbothioamide **3i**
^{1}H NMR (500 MHz, DMSO-d_6) δ 10.68 (1H, s, CONH-N), 10.58 (1H, s, NHN$\underline{H}$-CS), 10.51 (N$\underline{H}$NH-CS), 8.80 – 8.75 (2H, m, H2, H6), 8.67 (1H, s, CSN$\underline{H_2}$ *a*), 8.62 (1H, s, CSN$\underline{H_2}$ *b*), 7.80 – 7.75 (2H, m, H3, H5), 2.10 (3H, s, CH_3). ^{13}C NMR (126 MHz, DMSO) δ 179.88, 164.53, 163.23, 150.69, 140.36, 139.67, 121.45, 11.64. IR (ATR-Ge):624, 709, 749, 840, 897, 931, 998, 1046, 1072, 1123, 1258, 1338, 1367, 1424, 1489, 1507, 1537, 1621, 1668, 1693, 3037, 3204, 3296, 3374 cm^{-1}. Elemental analysis $C_{10}H_{12}N_6O_2S$ (280.31); calculated C, 42.85; H, 4.32; N, 29.98, found C, 42.99; H, 4.47; N, 30.15. White solid; yield 91%, mp: 250-251°C. R_f 0.2.

(*E*)-*N*′-[1-(2-Isonicotinoylhydrazineyl)-1-oxopropan-2-ylidene]isonicotinohydrazide **3j**

^{1}H NMR (500 MHz, DMSO-d_6) δ 11.23 (1H, s, CO-N<u>H</u>-NH-CO-Py), 10.76 (1H, s, CONH-N), 10.26 (1H, s, CO-NH-N<u>H</u>-CO-Py), 8.83 – 8.75 (4H, m, H2, H6, H2´, H6´), 7.81 – 7.76 (4H, m, H3, H5, H3´, H5´), 2.20 (3H, s, CH_3). ^{13}C NMR (126 MHz, DMSO) δ 164.17, 163.67, 150.67, 150.36, 139.68, 122.33, 121.55, 12.83. IR (ATR-Ge): 630, 689, 753, 790, 844, 874, 931, 1003, 1064, 1149, 1217, 1239, 1273, 1372, 1413, 1533, 1556, 1604, 1673, 1706, 3169 cm^{-1}. Elemental analysis $C_{15}H_{14}N_6O_3$ (326.32); calculated C, 55.21; H, 4.32; N, 25.75, found C, 55.33; H, 4.30; N, 25.88. Beige solid; yield 79%, mp: 143-145°C. R$_f$ 0.1.

(2*E*,2'*E*)-*N*,*N'*-(1,4-Phenylene)bis[2-(2-isonicotinoylhydrazineylidene)propanamide] **3k**
^{1}H NMR (500 MHz, TFA-d_1) δ 11.10 (2H, s, CONH-N), 10.50 (2H, s, NH-Ph), 9.25 – 9.15 (4H, m, H2, H6, H2´, H6´), 8.84 – 8.69 (4H, m, H3, H5, H3´, H5´), 7.86 – 7.76 (4H, m, phenylene linker), 2.54 (6H, s, CH_3, CH_3´). ^{13}C NMR (126 MHz, TFA) δ 156.33, 150.91, 145.00, 144.39, 135.76, 130.02, 128.94, 125.23, 12.64. IR (ATR-Ge): 637, 664, 700, 725, 753, 826, 845, 897, 1000, 1065, 1123, 1152, 1218, 1274, 1303, 1361, 1406, 1509, 1538, 1605, 1627, 1681, 1697, 2877, 3061, 3384 cm^{-1}. Elemental analysis $C_{24}H_{22}N_8O_4$ (486.49); calculated C, 59.25; H, 4.56; N, 23.03, found C, 59.39; H, 4.66; N, 23.16. Yellow solid; yield 99%, mp: 345-347°C. R$_f$ 0.1.

(*E*)-2-(2-Isonicotinoylhydrazineylidene)-*N*-(2-phenoxyphenyl)propanamide **3l**
^{1}H NMR (500 MHz, DMSO-d_6) δ 11.33 (1H, s, CONH-N), 9.84 (1H, s, NH-Ph), 8.78-8.74 (2H, m, H2, H6), 8.40 – 8.35 (1H, m, H6´), 7.82 – 7.74 (2H, m, H3, H5,), 7.44 – 6.90 (8H, m, H3´, H4´, H5´, H2´´, H3´´, H4´´, H5´´, H6´´), 2.20 (3H, s, CH_3). ^{13}C NMR (151 MHz, DMSO) δ 161.61, 159.39, 156.32, 150.22, 149.80, 145.99, 140.55, 130.24, 129.32, 124.45, 124.27, 124.15, 122.33, 120.30, 118.67, 11.56. IR (ATR-GE): 622, 670, 695, 756, 774, 822, 842, 869, 907, 993, 1101, 1147, 1164, 1206, 1246, 1279, 1318, 1382, 1411, 1474, 1521, 1671, 1684, 3068, 3181, 3379 cm^{-1}. Elemental analysis $C_{21}H_{18}N_4O_3$ (374.40); calculated C, 67.37; H, 4.85; N, 14.96, found C, 67.50; H, 4.99; N, 15.05. Beige solid; yield 52%, mp: 189-191°C. R$_f$ 0.5.

(*E*)-2-(2-Isonicotinoylhydrazineylidene)-*N*-(4-phenoxyphenyl)propanamide **3m**
^{1}H NMR (500 MHz, DMSO-d_6) δ 11.24 (1H, s, CONH-N), 10.08 (1H, s, NH-Ph), 8.78 (2H, d, J = 5.1 Hz, H2, H6), 7.83 – 7.73 (4H, m, H3, H5, H2´, H6´), 7.39 – 7.35 (2H, m, H3´´, H5´´), 7.11 (1H, tt, J = 7.3, 1.1 Hz, H4´´), 7.03 – 6.96 (4H, m, H3´, H5´, H2´´, H6´´), 2.22 (3H, s, CH_3). ^{13}C NMR (151 MHz, DMSO) δ 163.14, 157.64, 152.77, 150.63, 150.59, 150.56, 134.48, 130.45, 123.57, 122.55, 122.28, 119.74, 119.66, 118.51, 12.96. IR (ATR-Ge): 650, 692, 744, 756, 828, 842, 889, 928, 1023, 1072, 1149, 1221, 1257, 1269, 1293, 1369, 1415, 1489, 1506, 1533, 1563, 1598, 1622, 1657, 1683, 3034, 3253 cm^{-1}. Elemental analysis $C_{21}H_{18}N_4O_3$ (374.40); calculated C, 67.37; H, 4.85; N, 14.96, found C, 67.22; H, 5.00; N, 15.20. Pale yellow solid; yield 75%, mp: 172-173°C. R$_f$ 0.5.

(*E*)-*N*-[4-(4-Chlorophenoxy)phenyl]-2-(2-isonicotinoylhydrazineylidene)propanamide **3n**
^{1}H NMR (500 MHz, DMSO-d_6) δ 11.29 (1H, s, CONH-N), 10.11 (1H, s, NH-Ph), 8.76 (2H, d, J = 4.9 Hz, H2, H6), 7.78 – 7.73 (4H, m, H3, H5, H2´, H6´), 7.40 – 7.37 (2H, m, H3´, H5´), 7.02 (2H, d, J = 8.7 Hz, H3´´, H5´´), 6.98 (2H, d, J = 8.5 Hz, H2´´, H6´´), 2.21 (3H, s, CH_3). ^{13}C NMR (151 MHz, DMSO) δ 163.25, 156.75, 152.38, 150.67, 141.03, 134.97, 130.49, 130.35, 127.33, 122.51, 120.79, 120.38, 120.16, 120.04, 13.10. IR (ATR-Ge): 617, 722, 756, 817, 843, 853, 876, 929, 1015, 1092, 1103, 1149, 1232, 1254, 1268, 1370, 1411, 1489, 1511, 1527, 1601, 1656, 1687, 3278 cm^{-1}. Elemental analysis $C_{21}H_{17}ClN_4O_3$ (408.84); calculated C, 61.69; H, 4.19; N, 13.70, found C, 61.82; H, 4.01; N, 13.87. Beige solid; yield 66%, mp: 184-186°C. R$_f$ 0.6.

(*E*)-*N*-[3-Chloro-4-(4-chlorophenoxy)phenyl]-2-(2-isonicotinoylhydrazineylidene)propanamide **3o**

^{1}H NMR (500 MHz, DMSO-d_6) δ 11.40 (1H, s, CONH-N), 10.36 (1H, s, NH-Ph), 8.77 (2H, d, J = 5.0 Hz, H2, H6), 8.10 (1H, s, H2´), 7.82 (2H, d, J = 5.0 Hz, H3, H5), 7.79 – 7.67 (1H, m, H6´), 7.44 – 7.37 (2H, m, H3´´, H5´´), 7.22 – 7.17 (1H, m, H5´), 6.97 – 6.90 (2H, m, H2´´, H6´´), 2.24 (3H, s, CH_3). ^{13}C NMR (151 MHz, DMSO) δ 163.36, 156.16, 152.00, 150.24, 146.56, 140.99, 136.33, 130.00, 126.93, 125.13, 122.49, 122.31, 122.03, 121.91, 120.69, 118.50, 12.67. IR (ATR-Ge): 651, 706, 756, 824, 837, 843, 879, 937, 1012, 1057, 1149, 1219, 1260, 1271, 1369, 1402, 1487, 1526, 1591, 1662, 1686, 2930, 3270 cm^{-1}. Elemental analysis $C_{21}H_{16}Cl_2N_4O_3$ (443.28); calculated C, 56.90; H, 3.64; N, 12.64, found C, 56.99; H, 3.55; N, 12.54. Beige solid; yield 63%, mp: 151-152°C. R_f 0.5.

(*E*)-2-(2-Isonicotinoylhydrazineylidene)-*N*-{4-[(4-nitrophenyl)thio]phenyl}propanamide **3p**
^{1}H NMR (500 MHz, DMSO-d_6) δ 11.50 (1H, s, CONH-N), 10.41 (1H, s, NH-Ph), 8.78 (2H, d, J = 4.8 Hz, H2, H6), 8.16 – 8.09 (2H, m, H3´´, H5´´), 7.95 – 7.93 (2H, m, H2´, H6´), 7.84 (2H, d, J = 4.8 Hz, H3, H5), 7.58 – 7.52 (2H, m, H2´´, H6´´), 7.24 – 7.20 (2H, m H3´, H5´), 2.27 (3H, s, CH_3). ^{13}C NMR (151 MHz, DMSO) δ 163.53, 152.00, 151.65, 150.32, 148.77, 145.01, 140.60, 140.27, 135.99, 126.34, 124.49, 123.26, 122.36, 121.67, 13.03. IR (ATR-Ge): 651, 680, 741, 841, 854, 929, 1084, 1149, 1240, 1288, 1317, 1337, 1347, 1370, 1420, 1513, 1541, 1585, 1667, 1684, 3263 cm^{-1}. Elemental analysis $C_{21}H_{17}N_5O_4S$ (435.46); calculated C, 57.92; H, 3.94; N, 16.08, found C, 58.02 H, 4.15; N, 16.06. Beige solid; yield 98%, mp: 134-136°C. R_f 0.5.

(*E*)-*N*-[(1,1'-Biphenyl)-4-yl]-2-(2-isonicotinoylhydrazineylidene)propanamide **3q**
^{1}H NMR (500 MHz, DMSO-d_6) δ 11.28 (1H, s, CONH-N), 10.14 (1H, s, NH-Ph), 8.79 (2H, d, J = 5.0 Hz, H2, H6), 7.91 – 7.61 (8H, m, H3, H5, H2´, H3´, H5´, H6´, H2´´, H6´´), 7.44 (1H, t, J = 7.7 Hz, H3´´, H5´´), 7.33 (1H, t, J = 7.3 Hz, H4´´), 2.24 (3H, s, CH_3). ^{13}C NMR (151 MHz, DMSO) δ 163.53, 163.09, 151.54, 150.37, 140.77, 139.80, 137.87, 135.66, 129.12, 127.34, 127.14, 126.52, 122.26, 120.64, 12.74. IR (ATR-Ge): 634, 645, 717, 758, 769, 841, 929, 1006, 1076, 1146, 1242, 1271, 1293, 1372, 1408, 1486, 1528, 1575, 1590, 1607, 1676, 3036, 3225, 3360 cm^{-1}. Elemental analysis $C_{21}H_{18}N_4O_2$ (358.40); calculated C, 70.38; H, 5.06; N, 15.63, found C, 70.80; H, 5.00; N, 15.78. Pale yellow solid; yield 86%, mp: 243-245°C. R_f 0.5.

(*E*)-2-(2-Isonicotinoylhydrazineylidene)-*N*-(4-sulfamoylphenyl)acetamide **4a**
^{1}H NMR (500 MHz, DMSO-d_6) δ 12.54 (1H, s, CONH-N), 10.74 (1H, s, NH-Ph), 8.85 – 8.83 (2H, m, H2, H6), 8.00 (1H, s, N=CH), 7.95 – 7.93 (2H, m, H3, H5), 7.88 – 7.80 (4H, m, H2´, H3´, H5´, H6´), 7.31 (2H, s, NH_2). ^{13}C NMR (151 MHz, DMSO) δ 163.18, 162.02, 151.03, 143.09, 141.81, 140.28, 139.58, 127.17, 122.17, 120.23. IR (ATR-Ge): 686, 697, 747, 819, 840, 871, 905, 928, 1063, 1103, 1162, 1244, 1293, 1324, 1338, 1406, 1532, 1591, 1680, 3205, 3308 cm^{-1}. Elemental analysis $C_{14}H_{13}N_5O_4S$ (347.25); calculated C, 48.41; H, 3.77; N, 20.16, found C, 48.59; H, 3.98; N, 20.37. Pale yellow solid; yield 91%, mp: 251-252°C. R_f 0.2.

(*E*)-2-(2-Isonicotinoylhydrazineylidene)-*N*-{4-[*N*-(thiazol-2-yl)sulfamoyl]phenyl}acetamide **4c**
^{1}H NMR (500 MHz, DMSO-d_6) δ 12.65 (1H, s, SO_2NH-), 12.50 (1H, s, CONH-N), 10.71 (1H, s, NH-Ph), 8.82 (2H, d, J = 5.0 Hz, H2, H6), 7.97 (1H, s, N=CH), 7.90 (2H, d, J = 5.0 Hz, H3, H5), 7.86 – 7.81 (2H, m, H3´, H5´), 7.80 – 7.75 (2H, m, H2´, H6´), 7.25 (1H, d, J = 4.7 Hz, H4´´$_{Thz}$), 6.82 (1H, d, J = 4.6 Hz, H5´´$_{Thz}$). ^{13}C NMR (151 MHz, DMSO) δ 169.28, 163.15, 161.95, 151.03, 143.06, 142.06, 140.30, 137.67, 127.40, 125.01, 122.17, 120.20, 108.69. IR (ATR-Ge): 603, 636, 659, 685, 715, 754, 836, 856, 940, 1017, 1089, 1115, 1137, 1238, 1271, 1318, 1403, 1519, 1568, 1586, 1686, 1706, 3278 cm^{-1}. Elemental analysis $C_{17}H_{14}N_6O_4S_2$ (430.46); calculated C, 47.43; H, 3.28; N, 19.52, found C, 47.56; H, 3.11; N, 20.08. Pale yellow solid; yield 62%, mp: 243-245°C. R_f 0.1.

(*E*)-2-(2-Isonicotinoylhydrazineylidene)-*N*-{4-[*N*-(pyrimidin-2-yl)sulfamoyl]phenyl}acetamide **4e**
^{1}H NMR (500 MHz, DMSO-d_6) δ 12.50 (1H, s, SO_2NH-), 12.40 (1H, s, CONH-N), 10.75 (1H, s, NH-Ph), 8.84 – 8.76 (2H, m, H2, H6) 8.51 (2H, d, *J* = 4.8 Hz, H3´´, H5´´), 7.99 – 7.92 (3H, m, H3, H5, N=CH), 7.84 – 7.79 (2H, m, H3´, H5´), 7.76 – 7.72 (2H, m, H2´, H6´), 7.04 (3H, t, *J* = 4.8 Hz, H3´´). ^{13}C NMR (151 MHz, DMSO) δ 165.15, 162.05, 158.90, 157.45, 151.02, 142.97, 142.82, 135.40, 129.30, 122.16, 119.97, 116.33, 112.67. IR (ATR-Ge): 629, 640, 672, 752, 799, 841, 852, 942, 1055, 1073, 1095, 1167, 1247, 1269, 1326, 1406, 1448, 1489, 1526, 1542, 1588, 1665, 1688, 3040, 3226 cm^{-1}. Elemental analysis $C_{18}H_{15}N_7O_4S$ (425.42); calculated C, 50.82; H, 3.55; N, 23.05, found C, 51.00; H, 3.22; N, 23.40. Pale yellow solid; yield 68%, mp: 249-251°C. R_f 0.1.

(*E*)-*N*-{4-[*N*-(4,6-Dimethylpyrimidin-2-yl)sulfamoyl]phenyl}-2-(2-isonicotinoylhydrazineylidene)-acetamide **4f**
^{1}H NMR (500 MHz, DMSO-d_6) δ 12.53 (1H, s, SO_2NH-), 12.40 (1H, s, CONH-N), 10.78 (1H, s, NH-Ph), 8.80 (2H, d, *J* = 7.5 Hz, H2, H6), 7.97 – 7.59 (7H, m, H3, H6, H2´, H3´, H5, H6´, N=CH), 6.76 (1H, s, $CH_{Pyrimid}$), 2.26 (6H, s, 2×CH_3). ^{13}C NMR (151 MHz, DMSO) δ 165.00, 163.04, 156.71, 150.98, 143.01, 142.49, 140.61, 140.22, 130.83, 129.71, 122.17, 119.59, 112.38, 23.40. IR (ATR-Ge): 649, 688, 718, 779, 836, 902, 1037, 1079, 1125, 1141, 1521, 1291, 1314, 1402, 1429, 1531, 1592, 1630, 1675, 3200, 3368 cm^{-1}. Elemental analysis $C_{20}H_{19}N_7O_4S$ (453.48); calculated C, 52.97; H, 4.22; N, 21.62, found C, 53.11; H, 4.20; N, 21.77. Pale yellow solid; yield 69%, mp: 184-186°C. R_f 0.1.

(*E*)-*N*'-[2-(2-Isonicotinoylhydrazineyl)-2-oxoethylidene]isonicotinohydrazide **4j**
^{1}H NMR (500 MHz, DMSO-d_6) δ 11.40 (1H, s, CO-N<u>H</u>-NH-CO-Py), 10.60 (1H, s, CONH-N), 8.80 (2H, d *J* = 7.5 Hz, H2, H6), 8.75 (2H, d *J* = 7.5 Hz, H2´, H6´), 8.55 (1H, s, CO-NH-N<u>H</u>-CO-Py), 7.85 (2H, d *J* = 7.5 Hz, H3, H5), 7.80 (1H, s, N=CH), 7.75 (2H, d *J* = 7.5 Hz, H3´, H5´). ^{13}C NMR (151 MHz, DMSO) δ 160.52, 159.94, 150.86, 150.42, 149.72, 140.50, 121.90, 121.42, 121.16, 121.11. IR (ATR-Ge): 640, 677, 752, 778, 843, 1001, 1029, 1067, 1153, 1222, 1292, 1390, 1410, 1485, 1539, 1601, 1646, 3043, 3192 cm^{-1}. Elemental analysis $C_{14}H_{12}N_6O_3$ (312.29); calculated C, 53.85; H, 3.87; N, 26.91, found C, 53.99; H, 3.98; N, 27.15. Pale yellow solid; yield 74%, mp: 160-161°C. R_f 0.3.

2. ^{1}H and ^{13}C NMR spectra for key compounds

^{1}H spectrum (500 MHz) of (*E*)-2-(2-isonicotinoylhydrazineylidene)-*N*-(4-phenoxyphenyl)propanamide **3m**

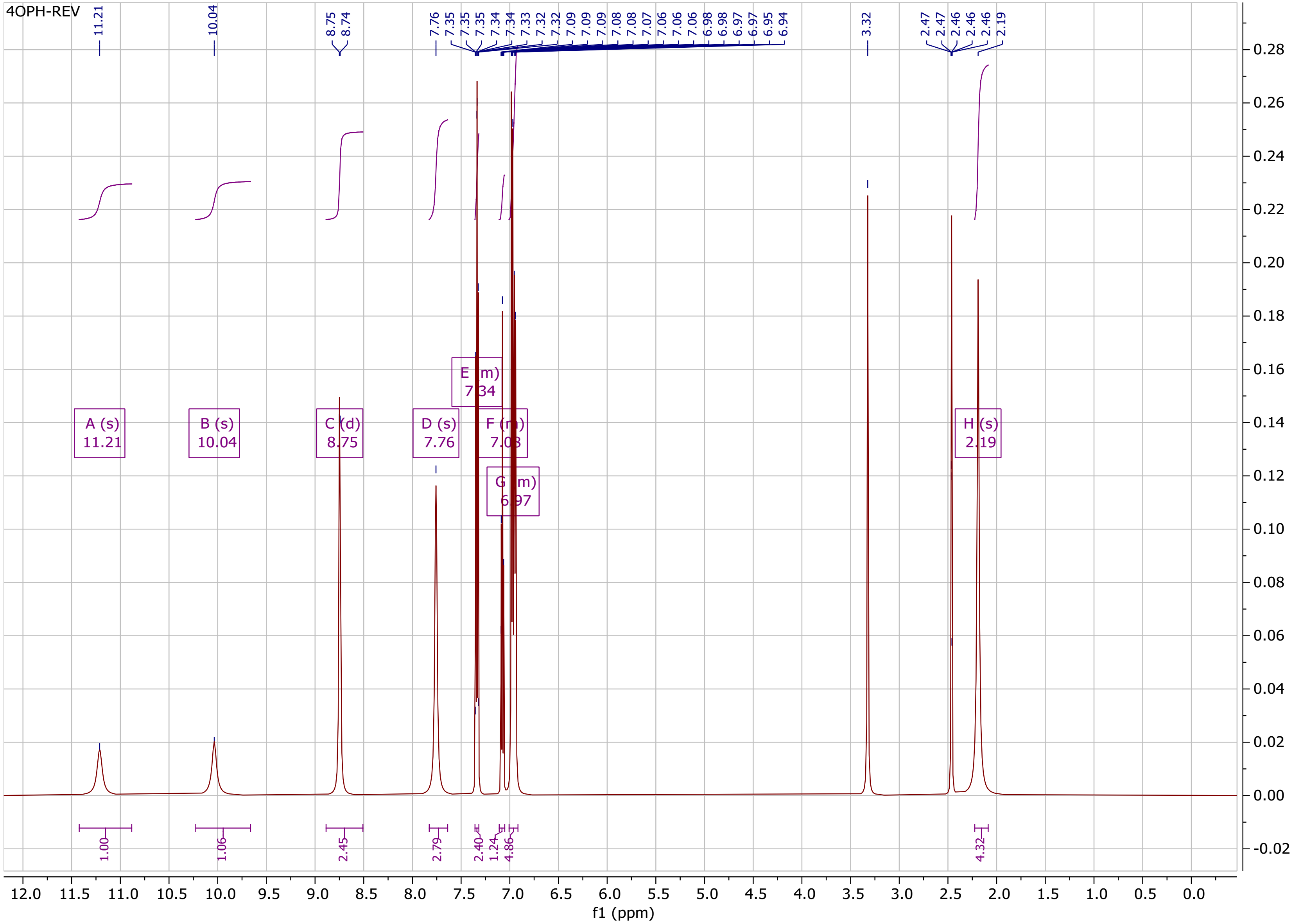

^{13}C spectrum (126 MHz) of (*E*)-2-(2-isonicotinoylhydrazineylidene)-*N*-(4-phenoxyphenyl)propanamide **3m**

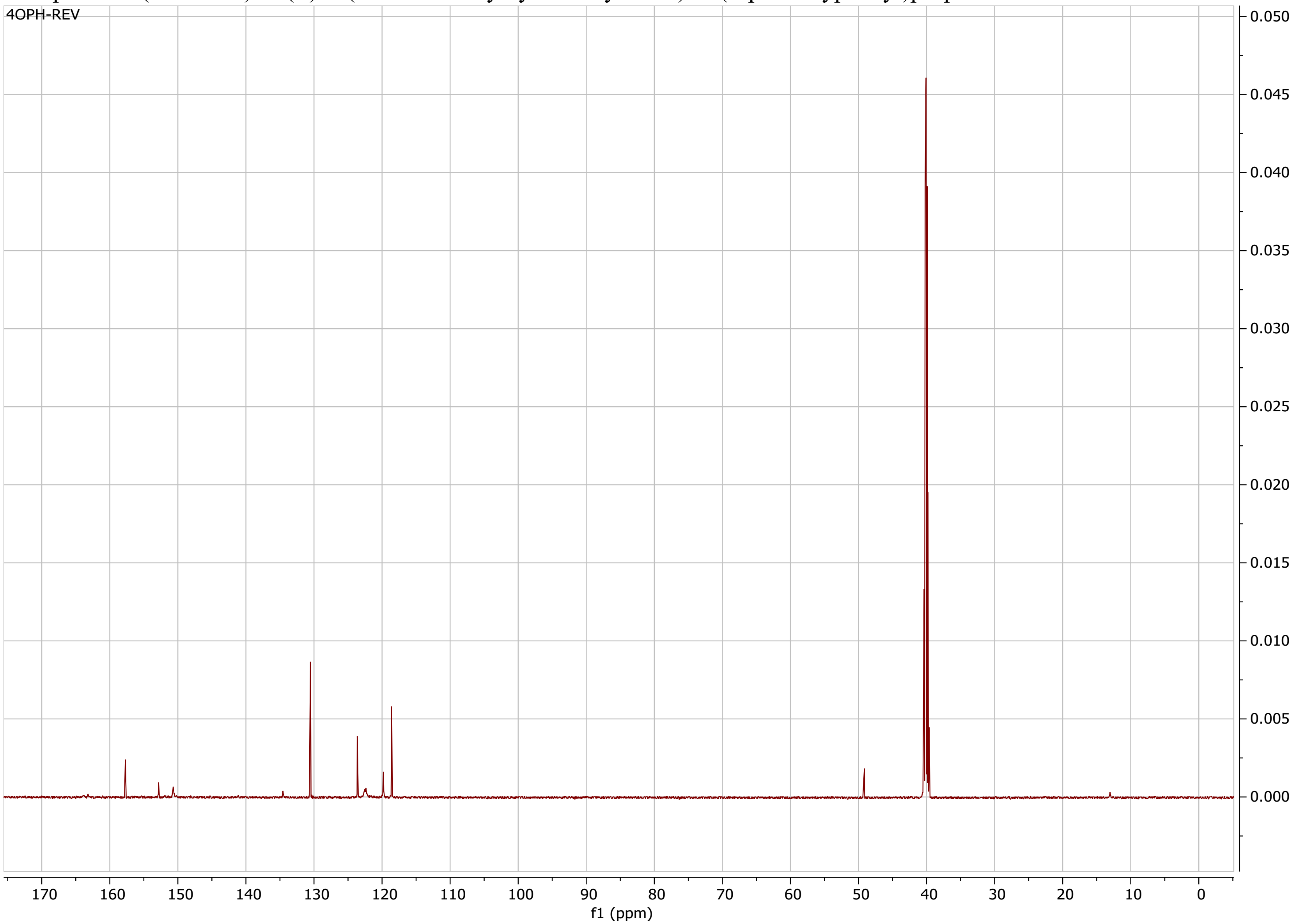

^{1}H spectrum (500 MHz) of (*E*)-2-(2-isonicotinoylhydrazineylidene)-*N*-{4-[(4-nitrophenyl)thio]phenyl}propanamide **3p**

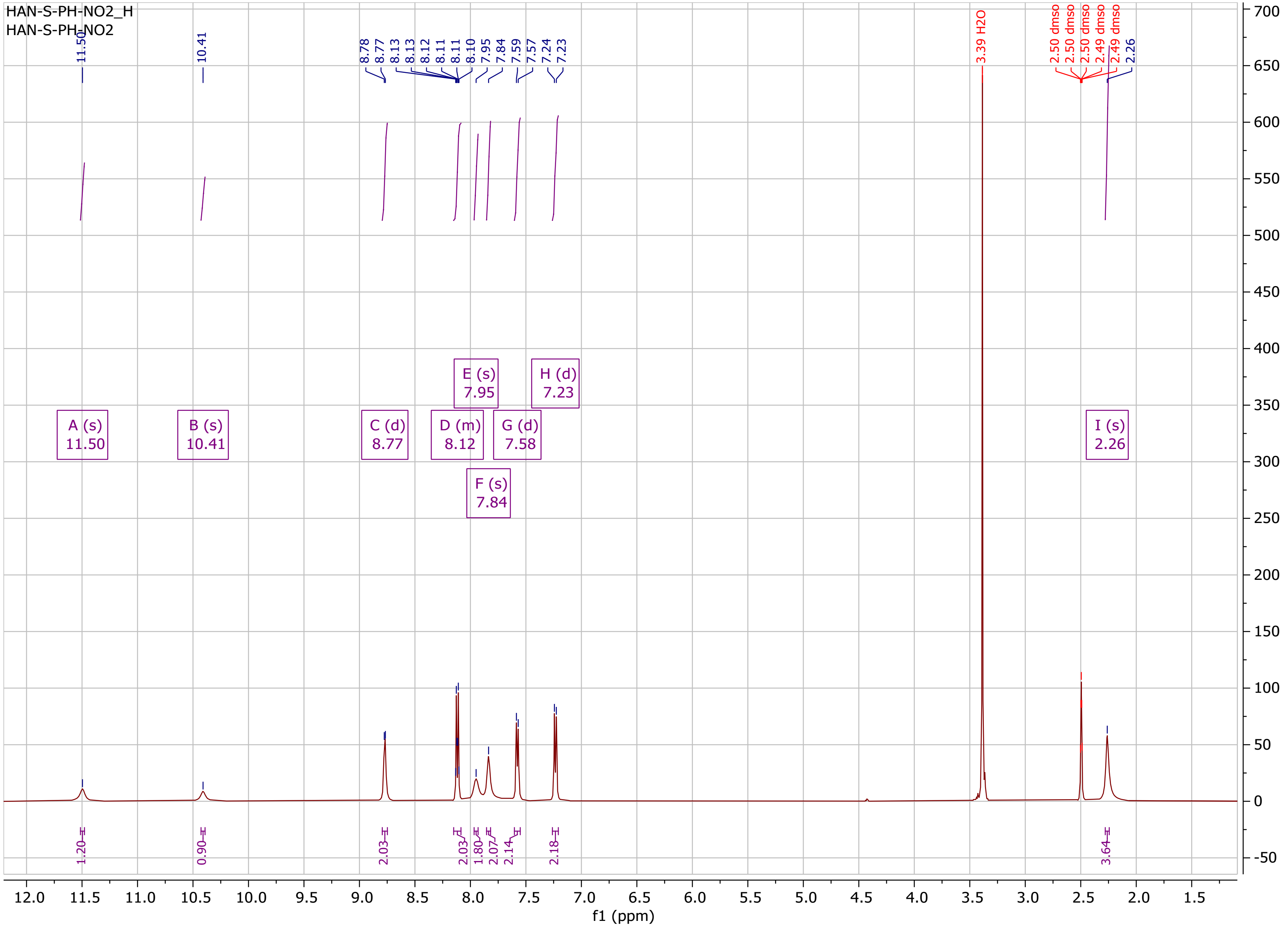

^{13}C spectrum (126 MHz) of (*E*)-2-(2-isonicotinoylhydrazineylidene)-*N*-{4-[(4-nitrophenyl)thio]phenyl}propanamide **3p**

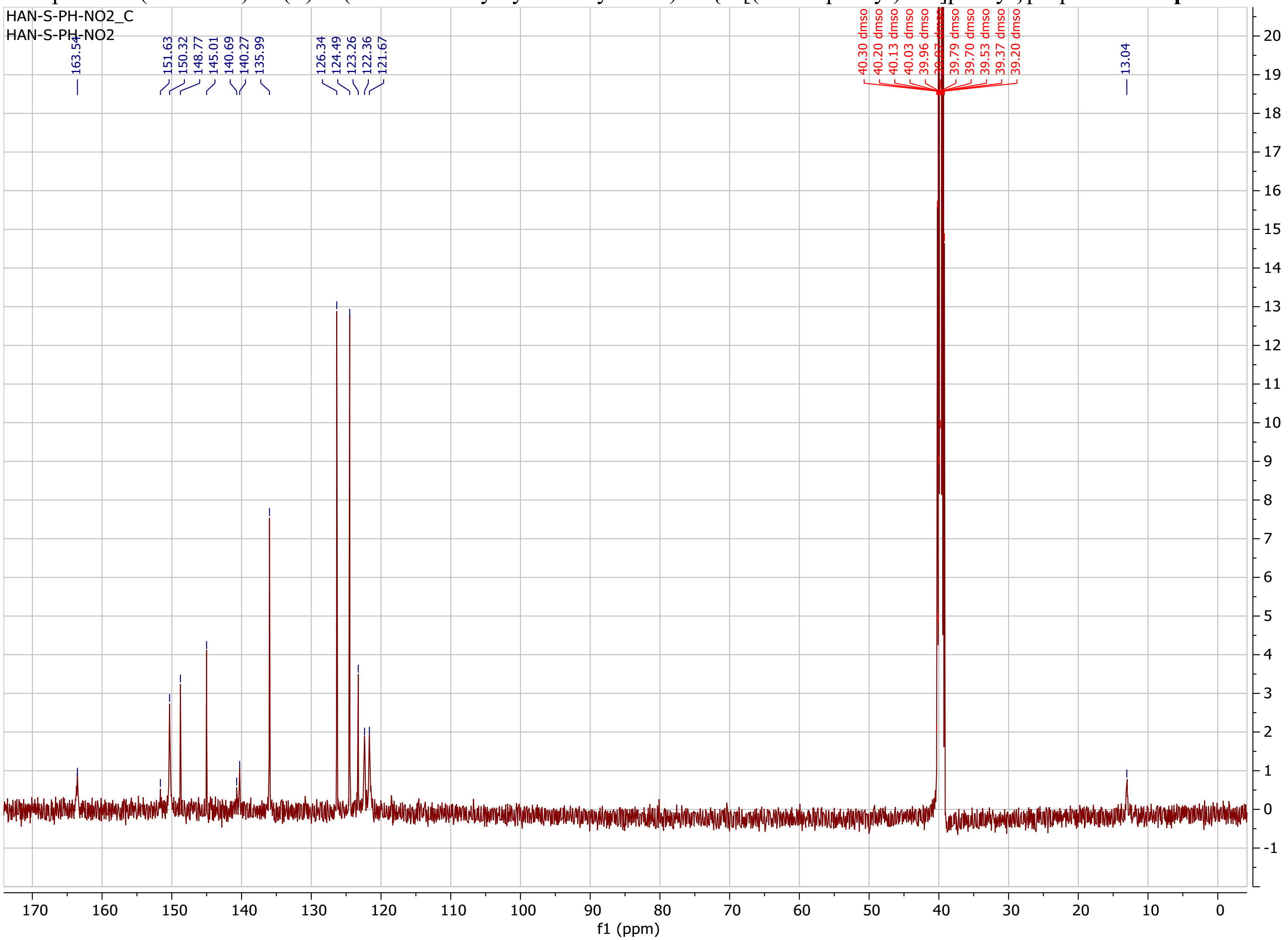

^{1}H spectrum (500 MHz) of (*E*)-*N*-[(1,1'-biphenyl)-4-yl]-2-(2-isonicotinoylhydrazineylidene)propanamide **3q**

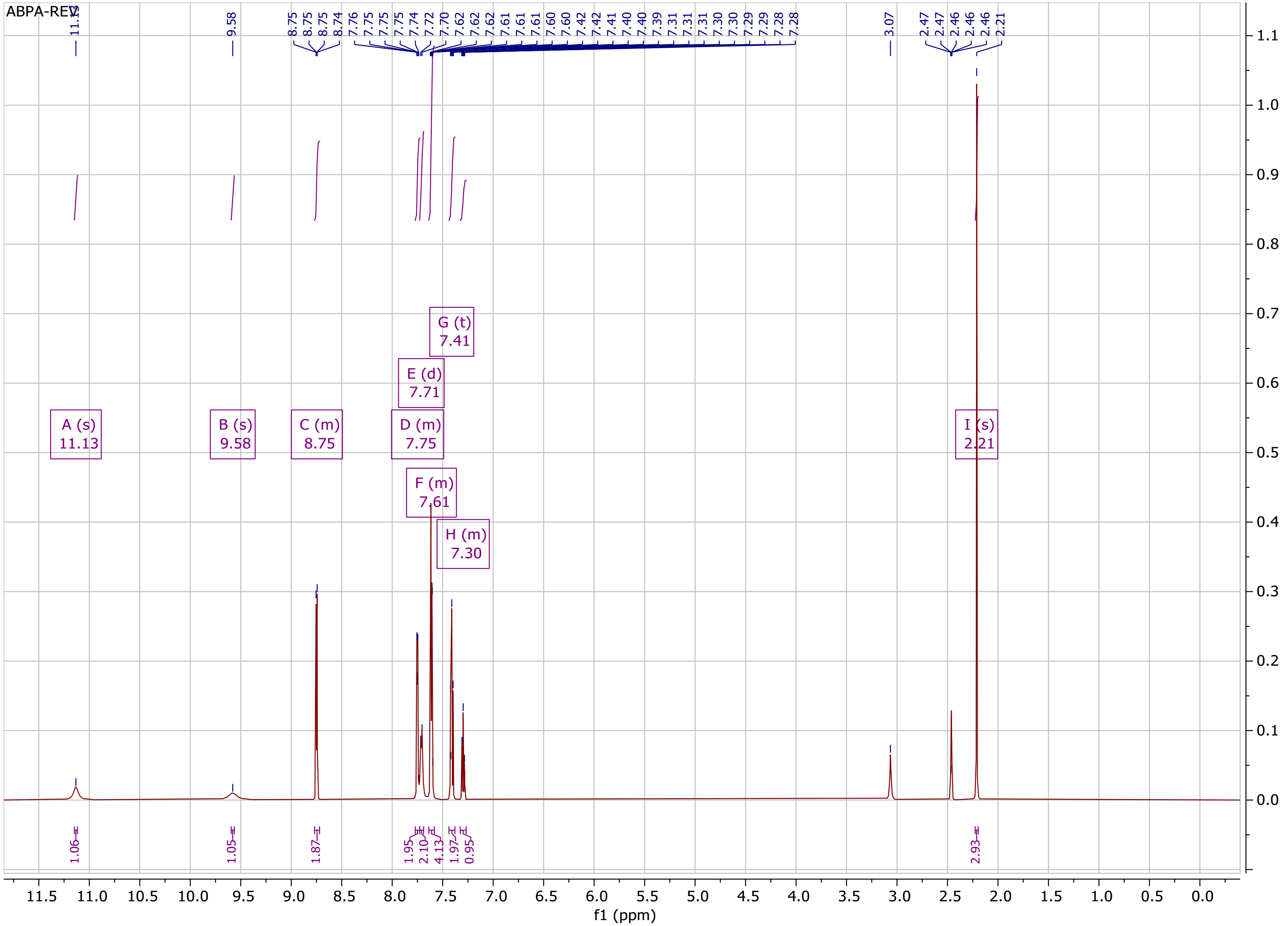

^{13}C spectrum (126 MHz) of (*E*)-*N*-[(1,1'-biphenyl)-4-yl]-2-(2-isonicotinoylhydrazineylidene)propanamide **3q**

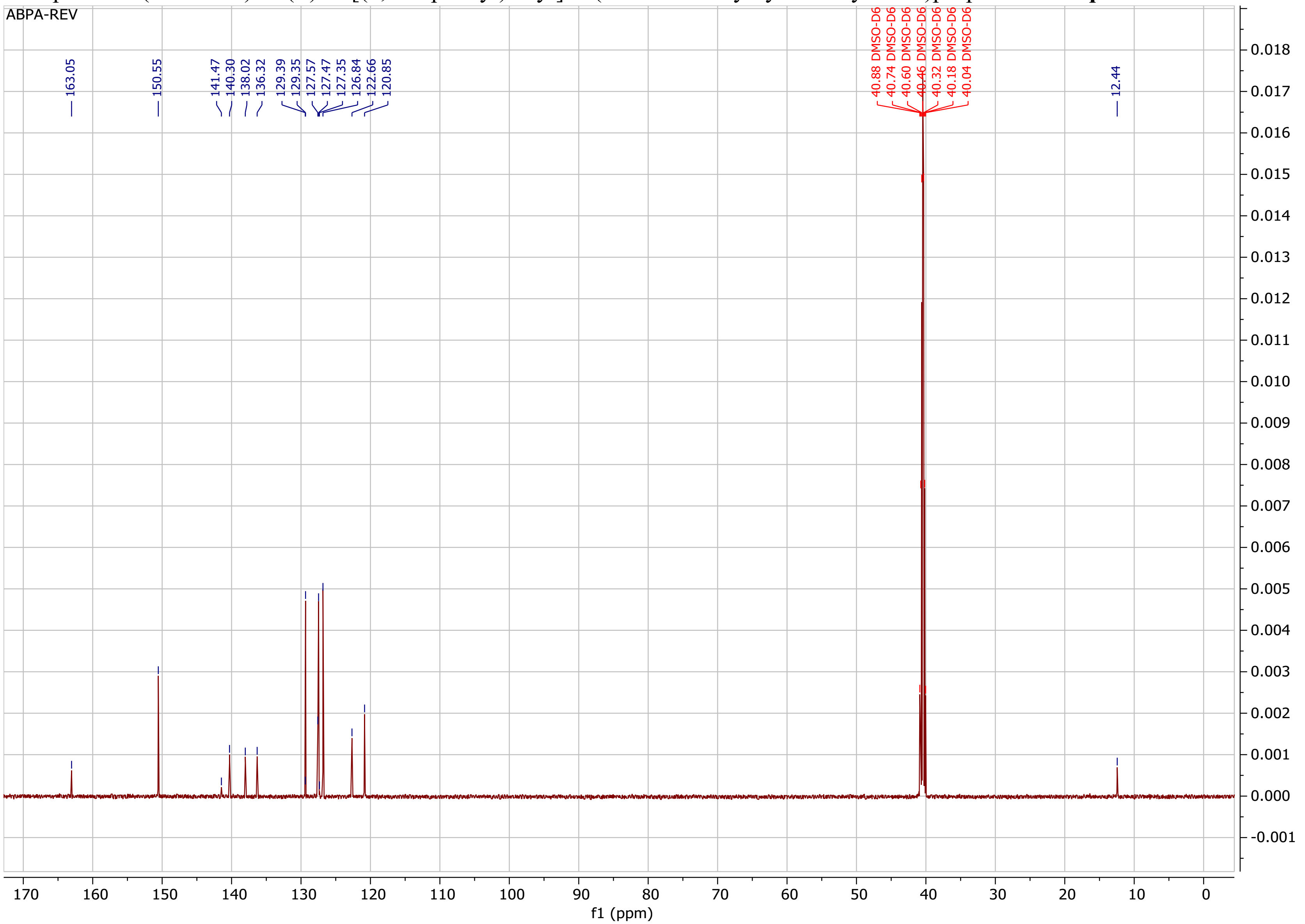

Supplement: MD-016-D4MD00663A-s001 [file MD-016-D4MD00663A-s001.pdf]
